# Structural basis for P-TEFb association with BRD4

**DOI:** 10.64898/2026.09.03.749171

**Published:** 2026-09-07

**Authors:** Abdallah A. Mohamed, Seychelle M. Vos

**Affiliations:** 1 Department of Biology, Massachusetts Institute of Technology; Cambridge, 02139, USA.; 2 Howard Hughes Medical Institute.

## Abstract

Cyclin-dependent kinases (CDK) dynamically regulate RNA Polymerase (Pol) II during each stage of transcription. Phosphorylation of RNA Pol II and associated transcription factors by CDKs enables stage-specific exchange of factors. CDK9 and a cyclin partner (CYCT1, T2 or K) comprise the Positive Transcription Elongation Factor-b (P-TEFb) complex, which is responsible for releasing RNA Pol II from promoter proximal pausing into processive elongation. P-TEFb activity is stimulated by bromodomain-containing Protein 4 (BRD4); however, the structural basis for BRD4 association with P-TEFb has remained unclear. Using cryogenic-electron microscopy, AlphaFold modeling, and biochemical probing, we show that the P-TEFb interacting domain of BRD4 (BRD4-PID) interacts with P-TEFb via the C-lobe of CDK9 and the second cyclin domain of CYCT1. The BRD4 binding surface on CDK9 is also used by super elongation complex component AFF4. Our analysis indicates that BRD4 may stimulate P-TEFb activity by remodeling the kinase αC helix relative to previous structures. Finally, P-TEFb activity is restricted when bound to the 7SK RNP complex. We show that in addition to BRD4-PID, the AFF4 N-terminus can release P-TEFb from the 7SK RNP complex. Together, these findings define how P-TEFb activity is regulated by its incorporation into distinct complexes.

## INTRODUCTION

RNA Polymerase (Pol) II transcribes most eukaryotic protein coding genes and is highly regulated to ensure appropriate gene expression.^1–3^ The association of distinct protein factors with RNA Pol II across the gene body modulates RNA Pol II activity.^1,3,4^ This differential factor association is partially regulated by the phosphorylation status of the largest RNA Pol II subunit, RPB1.^5,6^ RPB1 contains a C-terminal domain (CTD) composed of the heptapeptide repeat Y_1_S_2_P_3_T_4_S_5_P_6_S_7_.^1,5^ During transcription initiation, the initially hypophosphorylated CTD is modified by the kinase CDK7 to release initiation factors like Mediator from RNA Pol II and to allow for initial transcription.^7–9^ After promoter escape, metazoan RNA Pol II frequently pauses 25–100 bp from the transcription start site.^1^ This pause is initially induced by the underlying DNA sequence and is further modulated by transcription factors DRB sensitivity-inducing factor (DSIF) and negative elongation factor (NELF).^10–14^ Pause release and the assembly of productive elongation complexes requires the kinase positive transcription elongation factor b (P-TEFb).^15^ P-TEFb phosphorylates DSIF and NELF, as well as the RPB1 CTD and the RPB1 CTD linker.^13,15–19^ The phosphorylation of RNA Pol II and associated factors by P-TEFb allows for the displacement of NELF by elongation factors such as PAF1c and SPT6 and the transition of RNA Pol II from a paused state into a productive elongation state.^20,21^

P-TEFb is a heterodimer, composed of cyclin dependent kinase 9 (CDK9), and its predominant cyclin binding partner, cyclin-T1 (CYCT1), although it can associate with other cyclins, like cyclin-T2 and cyclin-K.^5,22–24^ CDK kinase activity is activated by the binding of its cyclin subunit and the phosphorylation of its T-loop, a conserved feature across CDKs.^25,26^ When CDKs are not bound to their cyclin partner, the T-loop is bound within the active site of the CDK, preventing substrate loading.^26,27^ Upon partner cyclin binding, the T-loop is rearranged, freeing the active site.^26,27^ CDK9’s activity is further regulated by the phosphorylation of T-loop residue Thr186 through CDK9’s autophosphorylation activity or by CDK-Activating Kinase (CAK), which is composed of CDK7, Cyclin H, and MAT1.^28,29^

P-TEFb activity is also regulated by its association with specific inactivating or activating protein binding partners. P-TEFb activity is inhibited when it is sequestered in the 7SK ribonucleoprotein (RNP) complex.^30,31^ Specifically, the HEXIM1 subunit of the 7SK RNP inhibits P-TEFb activity by associating with the CDK9 active site via its PYNT motif.^32,33^ In contrast, active P-TEFb containing complexes include the super elongation complex (SEC) or the bromodomain containing protein 4 (BRD4)–P-TEFb complex.^34–39^ The N-termini of SEC subunits AFF1/4 associate with P-TEFb.^40,41^ In addition to regulating transcription in uninfected cells, the SEC is involved in HIV-1 transcription, where AFF1/4 and HIV-1 Tat bind P-TEFb simultaneously.^22,38–40^ Structural and biochemical work have shown that the P-TEFb–AFF4–Tat complex enhances the association of HIV1 Tat with the HIV-1 Transactivation Response element (TAR) RNA hairpin associated with paused RNA Pol II.^41–44^ This interaction localizes P-TEFb to paused RNA Pol II to facilitate productive HIV-1 transcription.^39,42,45,46^

Human BRD4 is a 1362 amino acid protein that contains two N-terminal bromodomains that bind to acetylated histones, and an extra terminal (ET) domain that binds to other protein binding partners, such as JMJD6.^47–51^ The last 62 amino acids of the largely unstructured BRD4 C-terminus bind to P-TEFb and are referred to as the P-TEFb interaction domain (BRD4-PID).^50^ BRD4-PID can compete with P-TEFb binding to the 7SK RNP complex and stimulate P-TEFb’s kinase activity.^34–36,52^ Recent work has shown that the BRD4-PID is necessary for RNA Pol II to enter the gene body, suggesting that BRD4-PID is not only important for P-TEFb stimulation, but also important for transcription elongation.^53^ Biochemical work has been conducted to understand how BRD4-PID stimulates P-TEFb, identifying key motifs in BRD4-PID, namely BRD4-PID’s arginine rich motif (ARM) and leucine motif, that are important for the stimulation of P-TEFb activity.^52^ Finally, BRD4 and Tat have been reported to compete for P-TEFb binding, and inhibition of BRD4 by the bromodomain binding small molecule JQ1 leads to the transactivation of HIV-1.^54,55^ How HIV-1 Tat and BRD4-PID compete for P-TEFb association is not well understood.

We currently lack a structural understanding of how P-TEFb interacts with BRD4-PID and therefore the precise mechanism of how BRD4-PID activates P-TEFb. Furthermore, it is unknown how BRD4 or Tat release P-TEFb from HEXIM1. To address these questions, we have taken a structural and biochemical approach to characterize the P-TEFb–BRD4-PID complex. Our results indicate that P-TEFb uses an overlapping binding surface to interact with either BRD4 or AFF4 and show that both BRD4 and AFF4 can release P-TEFb from HEXIM1 inhibition. Together, these results provide a framework to understand how P-TEFb activity is regulated in distinct protein complexes.

## RESULTS AND DISCUSSION

### Biochemical characterization of the P-TEFb–BRD4-PID complex

To investigate how bromodomain containing protein 4 (BRD4) binds and stimulates positive transcription elongation factor b (P-TEFb), we recombinantly co-expressed and purified a human P-TEFb variant containing full length human CDK9 and a stable CYCT1 truncation (amino acids 1–272). We separately isolated the human BRD4 P-TEFb Interacting Domain (BRD4-PID) (amino acids 1300–1362) (Figure 1A and S1A). BRD4-PID and P-TEFb stably interacted in analytical size exclusion chromatography experiments (Figure S1B). We next determined the binding affinity of BRD4-PID for P-TEFb using bio-layer interferometry (BLI) and a biotinylated version of the BRD4-PID. The measured affinity of BRD4-PID for P-TEFb is a K_d_ of 100 nM ± 30 nM (Figure 1B). Our results differ from a previous publication where an affinity of 470 ± 140 nM was reported.^52^ The previous experiments were performed in a buffer containing 250 mM NaCl, whereas our buffer contained 100 mM NaCl. Salt sensitive association has been noted in prior coimmunoprecipitation experiments where stable P-TEFb-BRD4-PID interactions were only detected in buffers containing 200 mM or lower KCl concentrations.^34^

We next tested whether recombinantly purified BRD4-PID stimulated P-TEFb kinase activity. P-TEFb was incubated with increasing amounts of BRD4-PID in the presence of recombinantly expressed and purified human RPB1-CTD (amino acids 1593–1970) fused to maltose binding protein (MBP) and activity was monitored using a modified NADH-coupled ATPase assay (Figure 1C). Under our assay conditions, it is not feasible to regenerate the substrate (MBP-RPB1-CTD) meaning the assay was conducted under substrate depleting conditions (Figure 1C). Minimal background signal was observed from the MBP-RPB1-CTD on its own and the MBP-RPB1-CTD in the presence of BRD4-PID, indicating that ATPase activity is derived from P-TEFb’s kinase activity (Figure 1D). BRD4-PID stimulated P-TEFb ATPase activity in a titratable fashion as previously observed, resulting in a nearly two-fold increase in ATPase activity at the maximum concentration of BRD4-PID tested (Figure 1D).^52^

P-TEFb kinase activity can also be monitored by SDS-PAGE.^52^ The migration of hyper-phosphorylated MBP-RPB1-CTD (IIO) is retarded relative to unphosphorylated RPB1 CTD (IIA) in SDS-PAGE due to the presence of multiple negatively charged phosphate groups. Like the ATPase assays, P-TEFb incubated in the presence of a 100-fold molar excess of BRD4-PID produced a hyper-phosphorlyated CTD faster than isolated P-TEFb (Figures 1E and S1C). No detectable shift in MBP-RPB1-CTD was observed when the MBP-RPB1-CTD was only incubated with BRD4-PID (Figure S1D). Together, these results show that purified BRD4-PID associates with P-TEFb and stimulates P-TEFb kinase activity.

### Structure of the P-TEFb–BRD4-PID complex

We next visualized the P-TEFb–BRD4-PID complex using single particle cryo-EM. Purified P-TEFb and BRD4-PID along with ATPγS were incubated, applied to cryo-EM grids, and subjected to plunge freezing without crosslinking. Initial data processing indicated orientation bias in the sample. Thus, a subsequent data collection was conducted at a tilt angle of 15°. The tilted and non-tilted data sets were separately processed through particle picking and multiple rounds of 2D classification. Particles found in 2D classes with high resolution information and multiple views of the P-TEFb–BRD4-PID complex were selected, and the two particle sets were combined. *Ab-initio* classification was performed on the curated particle set and resulted in an initial map with density for P-TEFb and the BRD4-PID. Further classification and refinement resulted in a map that had a nominal resolution of 4.3 Å (gold standard FSC 0.143) (Figures 2, S2, S3, and Table 1). The model includes amino acids 1318–1358 of BRD4-PID, amino acids 17–321 of CDK9, and amino acids 10–248 of CYCT1, and density for ATPγS in the active site (Figures 1A, S3). The model shows good stereochemistry (Table 1).

Our model shows that the BRD4-PID binds to both CDK9 and CYCT1 via two α helices. BRD4-PID α-helix 1 (residues 1318–1342) interacts with the C-terminal lobe of CDK9 while α-helix 2 (residues 1349–1362) is sandwiched in between the CDK9 T-loop and CYCT1 and appears to contact the second cyclin domain of CYCT1 (Figures 1A and 2). Comparing our model to that of P-TEFb bound to ATP (PDB ID: 3BLQ), we observe that the buried surface area for P-TEFb increased by ~2-fold upon the binding of BRD4-PID, from an area of 886 Å^2^ in the ATP bound state to an area of 1683 Å^2^ in the BRD4-PID bound state. These observations are consistent with previous experiments that showed robust BRD4-PID association with the P-TEFb complex but not with CYCT1 on its own.^52^

### Structural comparisons between P-TEFb–BRD4-PID and other P-TEFb complexes

We next compared our P-TEFb–BRD4-PID model to previously solved X-ray crystal structures of P-TEFb bound to ATP or other protein factors. Each crystal structure was determined in a different space group, and thus crystal packing constraints could influence observed differences between structures. All P-TEFb structures solved to date are in an active kinase conformation. The P-TEFb–BRD4-PID model is also in an active conformation but is contracted relative to all other structures (Figure 3 and S4A–H).^29^ Specifically, BRD4-PID leads to the repositioning of the second cyclin domain in CYCT1, which is relatively immobile in all other reported structures (Figure 3A–B). The distance between CDK9 and the second cyclin domain of CYCT1 is 22.6 Å in an ATP bound structure of P-TEFb (PDB ID: 3BLQ) and is reduced to 17.2 Å in the presence of BRD4-PID (Figure S4E and S4H). Structures of HIV-1 Tat or AFF4-Tat bound P-TEFb retain the ~23 Å distance between CDK9 and CYCT1 as observed in the ATP bound structure (Figure S4F–H).^56 42^ The repositioning of the second cyclin domain is likely caused by the association of BRD4 α2 with CYCT1 (Figure 3A–B). Previous work implicated leucine residues (Leu1353 and Leu1354) in regulating P-TEFb activity.^52^ Substitution of BRD4 Leu1353 and Leu1354 to alanine resulted in reduced stimulation of P-TEFb activity.^52^ Our structural model suggests that BRD4 Leu1354 could coordinate binding to CYCT1 and likely contributes to the large scale conformational changes we observe in cyclin domain 2 (Figure 3C).

The αC kinase element undergoes massive conformational changes during kinase activation to facilitate formation of the kinase active site.^57^ The CDK9 αC is in an active conformation in all published structures, and its motion is coordinated with CYCT1 α helix H5.^29,41–43,56^ Protein factors HIV-1 Tat (PDB ID: 3MIA), AFF4 (PDB ID: 4IMY), or AFF4 and Tat (PDB ID: 4OGR) result in a shift in CYCT1 α helix H5, and a slight rotation in the CDK9 αC helix and/or the loop between β3 and αC to various degrees relative to P-TEFb bound to ATP (PDB ID: 3BLQ) (Figure 3D and S4A–D). BRD4-PID appears to exaggerate the changes in both elements. BRD4-PID appears to cause the CDK9 αC helix to move between 2–4 Å and the CYCT1 α helix H5 between 2–9 Å, depending on whether other protein factors, such as AFF4 or HIV-1 Tat, are bound to P-TEFb. In summary, BRD4 association with P-TEFb leads to more extensive conformational changes than observed in prior structures of P-TEFb.

### BRD4-PID competes with HIV-1 Tat binding on P-TEFb

Expression of HIV-1 is dependent on the recruitment of P-TEFb via HIV-1 Tat to the HIV-1 TAR RNA. BRD4-PID and HIV-1 Tat both stimulate P-TEFb activity^41,52^ and share sequence homology, including arginine rich and leucine motifs.^52^ Prior work showed that BRD4 residues 1209–1362 but not residues 1209–1328 displaced HIV-1 Tat from binding P-TEFb.^54^ Additionally, overexpression of BRD4-PID led to reduced expression of HIV-1 in J-Lat clone A2 cells.^54^ These findings suggested there may be a direct competition between BRD4 and HIV-1 Tat for P-TEFb.^54^ To examine this possibility, we compared our structure to a structure of P-TEFb bound to HIV-1 Tat.^56^ This analysis showed that BRD4 and HIV-1 Tat bound an overlapping binding surface on CYCT1. Specifically, an N-terminal loop in HIV-1 Tat (residues 7–13) clashes with BRD4-PID α2, providing an explanation for the previous biochemical and cell biological results (Figure S4I).

### The BRD4 PID–P-TEFb complex is mutually exclusive with the BRD4 PID-E2 complex

In addition to P-TEFb, BRD4-PID binds the human papillomavirus (HPV) E2 protein.^58,59^ The E2 protein among other roles is responsible for non-covalently tethering the papillomavirus genome to the host genome and for activating and repressing HPV gene expression.^58,59^ A X-ray crystal structure of BRD4-PID with the E2 protein showed that BRD4-PID contacted the E2 protein using α2 (residues 1349–1362).^60^ Although the BRD4-PID construct used in the E2 co-crystal structure contained the residues that interact with CDK9 (1325–1348), these residues are not resolved. Overlaying our structure with the BRD4-E2 structure showed that BRD4 would not be able to interact with both E2 and P-TEFb simultaneously (Figure S4J). This is in line with previous work that has shown that E2 binding to BRD4 inhibits the interaction between P-TEFb and BRD4.^61^ It is thought that this competition prevents the recruitment of P-TEFb to viral oncogenes E6/E7 of the integrated HPV genome to repress their expression.^61^

### P-TEFb binds BRD4 and AFF4 at a shared CDK9 interface using a conserved sequence motif

Active P-TEFb exists in at least two distinct complexes in the cell, those bound to BRD4 and those bound to the super elongation complex subunits AFF1/4. Published work indicates that BRD4 in complex with P-TEFb is required for proper basal transcription activation and in response to stresses such as hypoxia.^62^ AFF4 and the super elongation complex are associated with transcriptional activation in response to stress.^63^ Therefore, these different pools of active P-TEFb appear to regulate RNA polymerase II activity in a gene and context specific manner. To better understand these observations, we compared structures of P-TEFb bound to AFF4 with our P-TEFb–BRD4-PID structure. In all reported structures and in contrast to HIV-1 Tat and BRD4-PID, AFF4 does not come in proximity with CDK9’s T-loop. AFF4 additionally has no reported stimulatory function and may even slightly inhibit P-TEFb kinase activity.^41^

The N-terminal α0 of AFF4 interacts with the C-lobe of CDK9 (Figure 4A).^41–43^ This AFF4–CDK9 interaction was not extensively studied because it was thought to be a crystallization artifact at the unit cell interface of the AFF4–P-TEFb crystal structure. Furthermore, alanine scanning mutagenesis of AFF4 α0 had less impact on transcription activation in cells than AFF4 residues that interact with CYCT1.^41–43^ Notably, loss of the CDK9 binding interface reduced the affinity of AFF4 for P-TEFb.^41^

We next identified residues that may facilitate the interaction between BRD4-PID or AFF4 α0 and the CDK9 C-lobe. The residues that AFF4 and BRD4 use to interact with the C-lobe of CDK9 share sequence homology, with a sequence identity of 44% and a sequence similarity of 75%, suggesting that AFF4 and BRD4 use similar binding interactions (Figures 4A–B and S5A–B). Specifically, four residues on BRD4-PID Arg1325, Arg1329, Glu1332, and Arg1335, and their conserved counterparts on AFF4 Arg6, Arg10, Glu13, and Arg16, appear to coordinate binding to CDK9 residues Asp318, Trp316, Asn311, and Asp307, respectively (Figures 4A–B and S5C). BRD4 Arg1329 and Arg1335 have been implicated as a part of a larger cluster of basic residues on BRD4-PID that are involved in the stimulation of P-TEFb activity.^52^ To characterize the BRD4-CDK9 C-lobe interaction surface, we mutated interacting CDK9 residues to Asp318Ala, Trp316Val, Asn311Ala, and Asp307Ala (C-lobe mutant). BLI performed with the WT BRD4-PID and the C-lobe mutant showed no detectable binding of BRD4-PID (Figure 4C). Consistent with this ablation of binding between BRD4-PID and the C-lobe mutant, BRD4-PID was no longer able to stimulate the C-lobe mutant’s ATPase activity (Figure 4D). Importantly, the ATPase activity of the C-lobe mutant is similar to WT P-TEFb in the absence of BRD4-PID, indicating that the CDK9 C-lobe interface is required for BRD4-PID to bind to P-TEFb and not the general activity of P-TEFb.

### P-TEFb is released from the 7SK RNP complex by either BRD4-PID or AFF4

Up to 90% of P-TEFb in cells is found in the 7SK RNP complex, which inactivates P-TEFb kinase activity through interactions with 7SK protein HEXIM1/2.^30–32,64,65^ P-TEFb is released from the 7SK RNP complex by BRD4-PID and HIV-1 Tat *in vitro* and in cells.^52,66,67^ There are presently no experimentally derived structures of P-TEFb bound to HEXIM1. To understand how HIV-1 Tat, BRD4, or AFF4 bound P-TEFb may accommodate HEXIM1, we acquired an AlphaFold3 model of a HEXIM1-P-TEFb dimer. The 7SK RNP complex contains two HEXIM1 molecules that are dimerized by their C-terminal coiled-coil domains, and each HEXIM1 can bind a P-TEFb molecule (Figure S6).^68–70^ AFF4 and BRD4 appear to bind in an overlapping fashion with HEXIM1 on P-TEFb by competing with a predicted HEXIM1 α-helix spanning amino acids 257–279 (Figure 5A–B). This HEXIM1 helix is predicted to interact with CYCT1. Comparison of X-ray crystal structures of P-TEFb bound to both AFF4 and Tat (PDB ID: 4OGR) show that Tat shares a larger overlapping binding surface with HEXIM1 than AFF4, suggesting that AFF4 and Tat could work additively to release P-TEFb from HEXIM1.^42^

From our structural prediction of HEXIM1 bound P-TEFb, we hypothesized that AFF4 may release P-TEFb from the 7SK RNP complex. To test this, we used a modified version of the NADH-coupled ATPase assay to measure P-TEFb activity in the context of the 7SK complex (Figure 5C). Full-length HEXIM1 and stem loop 1 of the 7SK RNA (residues 24–86) that binds and releases an autoinhibitory patch on HEXIM1 were mixed with P-TEFb.^71^ Incubation of P-TEFb with the 7SK RNA on its own resulted in mild ATPase inhibition. In contrast, addition of HEXIM1 with or without stem loop 1 of the 7SK RNA and P-TEFb resulted in complete inhibition of P-TEFb’s apparent ATPase activity (Figure 5D). Titration of BRD4-PID into reactions containing P-TEFb, HEXIM1, and the 7SK RNA reversed this inhibition in a titratable manner, whereas reactions lacking P-TEFb but containing all other protein factors showed no change in ATPase activity (Figure 5D). This indicates that BRD4-PID activates P-TEFb, suggesting that BRD4-PID releases P-TEFb from the 7SK RNP complex. We next conducted these experiments with the N-terminus of AFF4 (amino acids 1–98). Like BRD4, AFF4 addition resulted in a titratable increase in P-TEFb activity (Figure 5D). Together these results show that both AFF4 and BRD4 can relieve P-TEFb ATPase inhibition that is mediated by HEXIM1 and the 7SK RNA. Our results suggest that the mechanism could involve competition with an overlapping surface on CYCT1 between HEXIM1 and BRD4/AFF4 (Figure 5 and S6).

## CONCLUSIONS

Here we structurally and biochemically characterized the interaction between P-TEFb and BRD4. We additionally investigated the regulatory interplay between P-TEFb binding partners BRD4, AFF4, HEXIM1, and HIV-1 Tat. AFF4 with HIV-1 Tat and BRD4 appear to stimulate P-TEFb activity by stabilizing P-TEFb in a more active conformation. Additionally, BRD4 and AFF4 associate with the C-lobe of CDK9 using a similar sequence motif and surface, and, thus, could not simultaneously bind P-TEFb. Finally, AFF4, like BRD4-PID and HIV-1 Tat, can release P-TEFb from the 7SK RNP complex. Complementary results describing the interaction of BRD4-PID to CYCT1 and CYCT2 were reported while we were in the final stages of preparing this manuscript.^72^

Proteins that associate with active P-TEFb can be classified into at least two categories: 1) those that stimulate P-TEFb’s kinase activity and release it from the 7SK RNP complex (*e.g*., HIV-1 Tat and BRD4), and 2) those that only release it from the 7SK RNP complex (e.g., AFF4). BRD4 and AFF4 differentially regulate gene expression.^62,63^ This separation of BRD4 and AFF4 function in cells could be linked to their differential influence on P-TEFb activity *in vitro*, where the fine-tuned expression of stressresponse related genes requires a less active P-TEFb bound to AFF4, but basal transcription requires more active P-TEFb-BRD4. This remains to be explored. It is unclear how different P-TEFb complexes could associate with RNA Pol II in a gene specific manner. Both the super elongation complex and BRD4 can associate with acetylated nucleosomes, and this could potentially facilitate P-TEFb recruitment to specific chromatin locations (Figure 6).^47,73–75^ Further work is needed to understand how P-TEFb is incorporated into specific complexes and brought to paused RNA Pol II.

## MATERIALS AND METHODS

### Cloning and Protein Expression

GST-P-TEFb and MBP-RPB1-CTD were cloned and expressed as previously described.^20^

MBP-P-TEFb and MBP-P-TEFb (C-lobe mutant) were cloned as follows: CDK9 and CDK9 (C-lobe mutant) plasmids were synthesized by Twist Bioscience. Full length CDK9 and CDK9 (C-lobe mutant) were inserted into a 438-A (AddGene: 55218) plasmid with an N-terminal TEV cleavable MBP tag. CYCT1 was inserted into a 438-A vector. CYCT1 and each respective CDK9 vector were combined into a pBig1a vector (AddGene: 80611) to produce the final P-TEFb (WT) and P-TEFb (C-lobe mutant) expression vector. The expression vectors were transformed into *E. coli* DH10EMBacY (Geneva Biotech) for bacmid generation. Positive colonies that contained the bacmid were isolated for amplification. Bacmids were isolated by alkaline lysis and ethanol precipitation. The bacmids were transfected into adherent Sf9 cells (Expression Systems) to produce V0 virus. V0 virus was amplified into V1 virus in Sf21 cells (Expression Systems). For large scale expressions, 1.2 L of Hi5 cells (Expression Systems) at a density of 1 million cells/mL were infected with V1 virus. After 72 hours cells were harvested by centrifugation and resuspended in lysis buffer (300 mM NaCl, 20 mM Na-HEPES pH 7.4, 10% (v/v) glycerol, 30 mM imidazole pH 8.0, and 1 mM DTT, 0.284 μg•mL^−1^ leupeptin, 1.37 μg•mL^−1^ pepstatin A, 0.17 mg•mL^−1^ PMSF, and 0.33 mg•mL^−1^ benzamidine). Resuspended cells were snap frozen in liquid nitrogen and stored at −80°C until purification.

BRD4-PID (BRD4 residues 1300–1362) was cloned into a 1C plasmid with a N-terminal MBP tag followed by a 6x His followed by a TEV cleavage site (Addgene: 29654). The Avi-tagged BRD4-PID were cloned using sequence and ligation independent cloning. The BRD4-PID constructs were transformed into Bl21 (DE3) RIL cells and grown at 37°C. Protein expression was induced with 0.5 mM IPTG when the cells reached an OD 600 nm of 0.6. Cells were grown for an additional 3 hours at 37°C. Cells were then harvested by centrifugation and resuspended in lysis buffer (300 mM NaCl, 20 mM Na-HEPES pH 7.4, 10% (v/v) glycerol, 30 mM imidazole pH 8.0, and 1 mM DTT, 0.284 μg•mL^−1^ leupeptin, 1.37 μg•mL^−1^ pepstatin A, 0.17 mg•mL^−1^ PMSF, and 0.33 mg•mL^−1^ benzamidine). Resuspended cells were snap frozen in liquid nitrogen and stored at −80°C until purification.

Full length HEXIM1 and AFF4 (residues 1–98) were cloned into a 1B plasmid with a N-terminal 6x His followed by a TEV cleavage site (AddGene: 29653). The HEXIM1 plasmid was transformed into Rosetta 2 pLysS cells and grown at 37°C. Protein expression was induced with 0.5 mM IPTG when the cells reached an OD 600 nm of 0.6. Cells were grown for an additional 3 hours at 30°C. Cells were then harvested by centrifugation and resuspended in lysis buffer (300 mM NaCl, 20 mM Na-HEPES pH 7.4, 10% (v/v) glycerol, 30 mM imidazole pH 8.0, and 1 mM DTT, 0.284 μg mL^−1^ leupeptin, 1.37 μg mL^−1^ pepstatin A, 0.17 mg•mL^−1^ PMSF, and 0.33 mg•mL^−1^ benzamidine). Resuspended cells were snap frozen in liquid nitrogen and stored at −80°C until purification. The AFF4 plasmid was transformed into Rosetta 2 pLysS cells and grown at 37°C. Protein expression was induced with 0.5 mM IPTG when the cells reached an OD 600 nm of 0.8. Cells were grown for an additional 16 hours at 16°C. Cells were then harvested by centrifugation and resuspended in lysis buffer (500 mM NaCl, 20 mM Na-HEPES pH 7.4, 10% (v/v) glycerol, 30 mM imidazole pH 8.0, and 1 mM DTT, 0.284 μg•mL^−1^ leupeptin, 1.37 μg•mL^−1^ pepstatin A, 0.17 mg•mL^−1^ PMSF, and 0.33 mg•mL^−1^ benzamidine). Resuspended cells were snap frozen in liquid nitrogen and stored at −80°C until purification.

### Protein Purification

All protein purification steps were performed at 4°C unless otherwise stated.

The GST tagged construct of P-TEFb, which was used for the cryo-EM sample, was purified from 2.8 liters of Hi5 expression. Cell pellets were thawed, lysed by sonication, and cleared by centrifugation. The clarified lysate was then filtered through a 5 μm syringe filter followed by a 0.45 μm syringe filter. The filtered lysate was applied to a 5 mL HisTrap HP column (Cytiva) that was equilibrated in lysis buffer (300 mM NaCl, 20 mM Na-HEPES pH 7.4, 10% (v/v) glycerol, 30 mM imidazole pH 8.0, and 1 mM DTT, 0.284 μg•mL^−1^ leupeptin, 1.37 μg•mL^−1^ pepstatin A, 0.17 mg•mL^−1^ PMSF, and 0.33 mg•mL^−1^ benzamidine). The HisTrap HP column was washed with ten column volumes of lysis buffer, followed by five column volumes of a high salt wash buffer (1000 mM NaCl, 20 mM Na-HEPES pH 7.4, 10% (v/v) glycerol, 30 mM imidazole pH 8.0, and 1 mM DTT) and five column volumes of 150 buffer (150 mM NaCl, 20 mM Na-HEPES pH 7.4, 10% (v/v) glycerol, 30 mM imidazole pH 8.0, and 1 mM DTT). A 5 mL HiTrap SP HP column (Cytiva) was equilibrated in nickel elution buffer (150 mM NaCl, 20 mM Na-HEPES pH 7.4, 10% (v/v) glycerol, 500 mM imidazole pH 8.0, and 1 mM DTT) and attached in-tandem with the HisTrap column. The protein was then eluted over a gradient from the 150-wash buffer to the nickel elution buffer over 30 minutes. After the elution concluded, the HisTrap column was removed and the HiTrap S column was washed with a S150 wash buffer (150 mM NaCl, 20 mM Na-HEPES pH 7.4, 10% (v/v) glycerol, and 1 mM DTT) for five column volumes. The protein was then eluted over a gradient from the S150 wash buffer with S1000 wash buffer (1000 mM NaCl, 20 mM Na-HEPES pH 7.4, 10% (v/v) glycerol, and 1 mM DTT) over 30 minutes. Peak fractions were pooled and mixed with 1.5 mg of 6x His-Tabacco Etch Virus (TEV) protease and dialyzed overnight against lysis buffer.^76^ The protein was then applied to a HisTrap equilibrated in lysis buffer to remove the 6xHis-TEV protease. Protein was concentrated to a 10 kDa MWCO Amicon Ultra Centrifugal Filter (Millipore) and applied to a HiLoad S200 16/1600 pg column equilibrated in SEC buffer (300 mM NaCl, 20 mM Na-HEPES pH 7.4, 10% (v/v) glycerol, and 1 mM DTT). Protein purity was assessed by SDS–PAGE followed by Coomassie staining. Peak fractions were concentrated in 10 kDa MWCO Amicon Ultra Centrifugal Filter (Millipore). The protein was stored in the SEC buffer. The protein was aliquoted, flash-frozen in liquid nitrogen, and stored at −80°C.

The MBP tagged P-TEFb (WT) and P-TEFb (C-lobe mutant), which were used for the biochemical assays, were purified as follows. 2.8 liters of Hi5 expression were used for each construct. Cell pellets were thawed, lysed by sonication, and cleared by centrifugation. The clarified lysate was then filtered through a 5 μm syringe filter followed by a 0.45 μm syringe filter. The filtered lysate was applied to a packed 10 mL amylose column (New England Biolabs) that was equilibrated in lysis buffer (300 mM NaCl, 20 mM Na-HEPES pH 7.4, 10% (v/v) glycerol, and 1 mM DTT, 0.284 μg•mL^−1^ leupeptin, 1.37 μg•mL^−1^ pepstatin A, 0.17 mg•mL^−1^ PMSF, and 0.33 mg•mL^−1^ benzamidine). The amylose column was washed with ten column volumes of lysis buffer, followed by five column volumes of high salt buffer (1000 mM NaCl, 20 mM Na-HEPES pH 7.4, 10% (v/v) glycerol, and 1mM DTT) and five column volumes lysis buffer. The protein was eluted using amylose elution buffer (300 mM NaCl, 20 mM Na-HEPES pH 7.4, 10% (v/v) glycerol, 116 mM maltose, and 1 mM DTT). Peak fractions were pooled and mixed with 1.5 mg of His_6_-TEV protease and dialyzed overnight against lysis buffer. The protein was then applied to a HiLoad S200 16/1600 pg column equilibrated in SEC buffer (300 mM NaCl, 20 mM Na-HEPES pH 7.4, 10% (v/v) glycerol, and 1 mM DTT). The peak fractions were pooled, concentrated in 10 kDa MWCO Amicon Ultra Centrifugal Filter (Millipore) to 5 mL and then applied to an amylose column equilibrated in SEC buffer to remove the cleaved MBP tag. Protein purity was assessed by SDS–PAGE followed by Coomassie staining. Peak fractions were concentrated in 10 kDa MWCO Amicon Ultra Centrifugal Filter (Millipore). The protein was stored in the SEC buffer. The protein was aliquoted, flash-frozen in liquid nitrogen, and stored at −80°C.

The BRD4-PID construct were purified as follows. Two liters of *E. coli* Bl21 (DE3) RIL were used for each protein preparation. Cell pellets were thawed, lysed by sonication, and cleared by centrifugation. The clarified lysate was then applied to a HisTrap equilibrated in lysis buffer (300 mM NaCl, 20 mM Na-HEPES pH 7.4, 10% (v/v) glycerol, and 1 mM DTT, 0.284 μg•mL^−1^ leupeptin, 1.37 μg•mL^−1^ pepstatin A, 0.17 mg•mL^−1^ PMSF, and 0.33 mg•mL^−1^ benzamidine). The column was washed with ten column volumes lysis buffer followed by five column volumes high salt buffer (1000 mM NaCl, 20 mM Na-HEPES pH 7.4, 10% (v/v) glycerol, and 1 mM DTT) and lysis buffer for five column volumes. The column was developed over 30-minutes in gradient containing nickel elution buffer (300 mM NaCl, 20 mM Na-HEPES pH 7.4, 10% (v/v) glycerol, 500 mM imidazole pH 8.0, and 1 mM DTT) at a flow rate of 1.5 mL/min. Peak fractions were pooled and mixed with 1.5 mg of 6x His-TEV protease and dialyzed overnight against lysis buffer. The protein was then applied to a HisTrap equilibrated in lysis buffer to remove the 6x His-TEV protease, uncleaved protein, and the 6x His-MBP tag. Protein was concentrated in a 3 kDa MWCO Amicon Ultra Centrifugal Filter (Millipore) and applied to a HiLoad S75 16/1600 pg column equilibrated in SEC buffer (300 mM NaCl, 20 mM Na-HEPES pH 7.4, 10% (v/v) glycerol, and 1 mM DTT). Protein purity was assessed by SDS–PAGE and Coomassie staining. Peak fractions were concentrated in a 3 kDa MWCO Amicon Ultra Centrifugal Filter (Millipore). The protein was stored in the SEC buffer. The protein was aliquoted, flash-frozen in liquid nitrogen, and stored at −80°C.

The Avi-BRD4-PID was purified like BRD4-PID except for the following modifications. After eluting from the HisTrap column, proteins were concentrated in 10 kDa MWCO Amicon Ultra Centrifugal Filter (Millipore) to 1 mL and incubated with BirA-6x His, 500 μM biotin, 1.0 mg TEV protease, 10 mM ATP pH 7.0, and 10 mM MgCl_2_ for 2 hours at 30°C. The reaction was incubated at 4°C overnight. The protein was then applied to a HisTrap column in tandem with a HiLoad S75 16/1600 pg column equilibrated in SEC buffer. The AFF4 (1–98) construct was expressed in two liters of *E. coli* Rosetta 2 (DE3) pLysS. Cell pellets were thawed, lysed by sonication, and cleared by centrifugation. The clarified lysate was then applied to a 5 mL HisTrap column equilibrated in lysis buffer (500 mM NaCl, 20 mM Na-HEPES pH 7.4, 10% (v/v) glycerol, and 1 mM DTT, 0.284 μg•mL^−1^ leupeptin, 1.37 μg•mL^−1^ pepstatin A, 0.17 mg•mL^−1^ PMSF, and 0.33 mg•mL^−1^ benzamidine). The column was washed with ten column volumes lysis buffer followed by five column volumes high salt buffer (1000 mM NaCl, 20 mM Na-HEPES pH 7.4, 10% (v/v) glycerol, and 1 mM DTT) and five column volumes lysis buffer. The column was developed over a 30-minute gradient using nickel elution buffer (500 mM NaCl, 20 mM Na-HEPES pH 7.4, 10% (v/v) glycerol, 500 mM imidazole pH 8.0, and 1 mM DTT). Peak fractions were pooled and mixed with 1.5 mg of His_6_-TEV protease and dialyzed overnight against lysis buffer. The protein was then applied to a HisTrap column equilibrated in lysis buffer to remove the His_6_-TEV protease, uncleaved protein, and the 6x His-MBP tag. Protein was concentrated in a 3 kDa MWCO Amicon Ultra Centrifugal Filter (Millipore) and applied to a HiLoad S75 16/1600 pg column equilibrated in SEC buffer (300 mM NaCl, 20 mM Na-HEPES pH 7.4, 10% (v/v) glycerol, and 1 mM DTT). Protein purity was assessed by SDS–PAGE followed by Coomassie staining. Peak fractions were concentrated in 3 kDa MWCO Amicon Ultra Centrifugal Filters (Millipore). The protein was aliquoted, flash-frozen in liquid nitrogen, and stored at −80°C.

HEXIM1 was purified from four liters of *E. coli* Rosetta 2 (DE3) pLysS. Cell pellets were thawed and lysed by sonication. After sonication, polyethylenimine was added to the lysate to a final concentration of 0.2% (w/v) while the lysate was stirring. The lysate was left to incubate for five minutes while stirring before it was cleared by centrifugation. The clarified lysate was then applied to a 5 mL HisTrap column equilibrated in lysis buffer (300 mM NaCl, 20 mM Na-HEPES pH 7.4, 10% (v/v) glycerol, and 1 mM DTT, 0.284 μg•mL^−1^ leupeptin, 1.37 μg•mL^−1^ pepstatin A, 0.17 mg•mL^−1^ PMSF, and 0.33 mg•mL^−1^ benzamidine). The column was washed with ten column volumes lysis buffer followed by five column volumes high salt buffer (1000 mM NaCl, 20 mM Na-HEPES pH 7.4, 10% (v/v) glycerol, and 1 mM DTT) and lysis buffer for five column volumes. The column was developed over a 30-minute gradient against nickel elution buffer (300 mM NaCl, 20 mM Na-HEPES pH 7.4, 10% (v/v) glycerol, 500 mM imidazole pH 8.0, and 1 mM DTT). Peak fractions were pooled and mixed with 1.5 mg of 6x His-TEV protease and dialyzed overnight against lysis buffer. The protein was then applied to a HisTrap equilibrated in lysis buffer to remove the His_6_-TEV protease, uncleaved protein, and the His_6_ tag. Protein was concentrated in a 30 kDa MWCO Amicon Ultra Centrifugal Filter (Millipore) and applied to a HiLoad S200 16/1600 pg column equilibrated in SEC buffer (300 mM NaCl, 20 mM Na-HEPES pH 7.4, 10% (v/v) glycerol, and 1 mM DTT). Protein purity was assessed by SDS–PAGE followed by Coomassie staining. Peak fractions were concentrated in 30 kDa MWCO Amicon Ultra Centrifugal Filter (Millipore). The protein was stored in the SEC buffer. The protein was aliquoted, flash-frozen in liquid nitrogen, and stored at −80°C.

The RPB1-CTD construct was purified from six liters of *E. coli* Bl21 (DE3) RIL. Cell pellets were thawed, lysed by sonication, and cleared by centrifugation. The clarified lysate was applied to a 5 mL HisTrap column equilibrated in lysis buffer (300 mM NaCl, 20 mM Na-HEPES pH 7.4, 10% (v/v) glycerol, and 1 mM DTT, 0.284 μg•mL^−1^ leupeptin, 1.37 μg•mL^−1^ pepstatin A, 0.17 mg•mL^−1^ PMSF, and 0.33 mg•mL^−1^ benzamidine). The column was washed with ten column volumes lysis buffer followed by five column volumes high salt buffer (1000 mM NaCl, 20 mM Na-HEPES pH 7.4, 10% (v/v) glycerol, and 1 mM DTT) and lysis buffer for five column volumes. A column packed with 10 mL of amylose resin was equilibrated in lysis buffer was attached to the base of the HisTrap column. The HisTrap column was developed over a 30-minute gradient against nickel elution buffer (300 mM NaCl, 20 mM Na-HEPES pH 7.4, 10% (v/v) glycerol, 500 mM imidazole pH 8.0, and 1 mM DTT). The HisTrap column was removed, and the amylose column was washed with five column volumes of lysis buffer. The protein was eluted using amylose elution buffer (300 mM NaCl, 20 mM Na-HEPES pH 7.4, 10% (v/v) glycerol, 116 mM maltose, and 1 mM DTT). Peak fractions were concentrated in a 30 kDa MWCO Amicon Ultra Centrifugal Filter (Millipore). The protein was then applied to a HiLoad S200 16/1600 pg column equilibrated in SEC buffer (300 mM NaCl, 20 mM Na-HEPES pH 7.4, 10% (v/v) glycerol, and 1 mM DTT). Protein purity was assessed by SDS–PAGE followed by Coomassie staining. Peak fractions were concentrated in a 30 kDa MWCO Amicon Ultra Centrifugal Filter (Millipore). The protein was stored in the SEC buffer. The protein was aliquoted, flash-frozen in liquid nitrogen, and stored at −80°C.

### Analytical Size-Exclusion Chromatography

P-TEFb (5 μM) and BRD4-PID (10 μM) were incubated in a final buffer containing 100 mM NaCl, 20 mM Na-HEPES pH 7.4, 10% (v/v) glycerol, and 1 mM DTT and incubated for 30 minutes at 30°C. The complex was applied to a Superdex 200 3.2/300 column equilibrated in complex buffer on an Äkta Micro purification system (Cytvia) at 4 °C (100 mM NaCl, 20 mM Na-HEPES pH 7.4, 4% (v/v) glycerol, 3 mM MgCl_2_, and 1 mM DTT). Additionally, P-TEFb and BRD4-PID were applied to a Superdex 200 3.2/300 column equilibrated in complex buffer on an Äkta Micro purification system (Cytvia) at 4 °C on their own. Peak fractions were analyzed by SDS–PAGE using a 4–12% gradient gel followed by Coomassie staining.

### Biolayer interferometry

All protein used for biolayer interferometry (BLI) was dialyzed against 500 mL of BLI assay buffer (20 mM Na-HEPES pH 7.4, 100 mM NaCl, 0.02% Tween-20, 1 mM DTT) for at least four hours at 4°C. Octet SA biosensors (Satorius) coated with streptavidin were incubated in BLI assay buffer for 10 minutes at 30 °C. The SA biosensors were incubated with BLI buffer for 60 seconds, then moved to wells of 200 μL of 10 nM of Avi-BRD4-PID for 300 seconds. The SA biosensors were then incubated with serially diluted P-TEFb (WT) or P-TEFb (C-lobe mutant) (Dilution Series 0–1000 nM) for 300 seconds to measure association. The SA biosensors were then incubated with BLI buffer for 600 seconds to measure dissociation for the Avi-BRD4-PID construct. Throughout the experiment the plate was shaking at 1000 rpm and kept at 30 °C. A set of SA biosensors that were not incubated with Avi-tagged protein were also applied to the P-TEFb titration series to account for nonspecific binding. The signal was subtracted from the no Avi-tagged protein and the 0 nM P-TEFb control for the association and dissociation curve. The experiments were performed in triplicate. Binding curves were fit for each replicate using the 333 nM, 111 nM, and 37 nM concentrations, due to having a r^2^ fit value of 0.9 or greater, in GraphPad Prims Version 10 and the average and standard deviation of the K_d_ from each binding replicate were determined and reported.

### NADH-coupled ATPase Assay

A modified NADH coupled ATPase assay was used to measure relative rates of ATP hydrolysis of P-TEFb in the presence BRD4-PID as previously described.^20,77^ P-TEFb was kept at 0.3 μM in a final buffer containing 3 mM MgCl_2,_ 0.1 mM NADH, 0.4% (w/v) pyruvate kinase/lactate dehydrogenase, 1 mM phosphoenolpyruvate, 100 mM NaCl, 20 mM Na-HEPES pH 7.4, 10% (v/v) glycerol and 1 mM DTT. BRD4-PID was titrated (0–10 μM). 5 μM of MBP-RPB1-CTD was used. Samples were incubated for 5 min at 30 °C before adding 1 mM ATP pH 7.0. The absorption at 340 nm was measured in 384-well, clear flat bottom, square well plates (Grenier: 781101) over 60 min at 30 °C in a Tecan Spark plate reader. The rate of change in absorbance at 340 nm over time was determined from the linear region of the resulting absorbance curves. All experiments were performed three times.

For the 7SK release assays, experiments were performed as above except, complexes containing P-TEFb, P-TEFb and the 7SK RNA, P-TEFb and HEXIM1, and P-TEFb, the 7SK RNA, and HEXIM1, were first incubated in a buffer containing 100 mM NaCl, 20 mM Na-HEPES pH 7.4, 10% glycerol, 3 mM MgCl_2_, and 1 mM DTT for 30 minutes at 30 °C, before being used in the assays at a final concentration of 0.3 μM. BRD4-PID and AFF4 (1–98) were titrated (0–10 μM). The 7SK RNA was synthesized by IDT and contained a FAM label on the 5’ end of the RNA and encompassed residues 25–86 of the 7SK RNA: RNA (5'−6-FAM-CAU CUG UCA CCC CAU UGA UCG CCA GGG UUG AUU CGG CUG AUC UGG CUG GCU AGG CGG UG-3').

### Gel-based P-TEFb Activity Assay

P-TEFb (0.1 μM) was incubated with 10 μM BRD4-PID and 5 μM of MBP-RPB1-CTD in a final buffer containing 3 mM MgCl_2,_, 100 mM NaCl, 20 mM Na-HEPES pH 7.4, 10% (v/v) glycerol and 1 mM DTT for 5 minutes at 30 °C before adding 1 mM ATP pH 7.0 in final volume of 50 μL. 5 μL of the reaction was removed after 0, 1, 5, 15, 30, 60, and 120 minutes after ATP addition and mixed with 5 μL of 2x SDS loading dye buffer (100 mM Tris-HCl pH 6.8, 4% w/v SDS, 20% (v/v) glycerol, 0.01% (w/v) bromophenol blue, 10 mM 2-Mercaptoethanol). This experiment was also done with 0.3 μM P-TEFb incubated with 5 μM of MBP-RPB1-CTD and with 10 μM BRD4-PID incubated with 5 μM of MBP-RPB1-CTD as controls. Time points were analyzed by SDS–PAGE using 4–12% gradient SDS-PAGE gels followed by Coomassie staining. Experiments were performed three times.

### AlphaFold Modeling

AlphaFold 3 modeling was performed on the online AlphaFold server.^78^ For the P-TEFb-PID AlphaFold 3 model CDK9 residues 1–330 were co-folded with CYCT1 residues 1–259 and BRD4 residues 1325–1362. For the P-TEFb–HEXIM1 AlphaFold 3 model, two molecules of CDK9 residues 1–372 were co-folded with two molecules of CYCT-1 residues 1–272 and two molecules of HEXIM1 residues 1–359. Models were visualized in ChimeraX v1.10.1.

### Cryo-EM Sample Preparation

P-TEFb (4 μM) was incubated with 20 μM BRD4-PID and 2 mM ATPγS in a final buffer of 100mM NaCl, 20mM Na-HEPES pH 7.4, 3.5% (v/v) glycerol, 6 mM MgCl_2_, and 1 mM DTT. The sample was incubated for 30 minutes at 30 °C shaking at 300 rpm. The sample was applied to R2/2 UltraAuFoil grids (Quantifoil) that were glow discharged for 2 minutes using a K100X glow discharger before 2 μL of sample was applied to each side of the grid. The sample was incubated for 4 seconds and blotted for 1 second. Vitrification was done by plunging the grids into liquid ethane using a Vitrobot Mark IV (FEI Company) maintained at 5 °C and 100% humidity.

### Cryo-EM Data Collection and Processing

Cryo-EM data were collected on a FEI Titan Krios operated at 300 keV. A K3 summit direct detector with a GIF quantum energy filter (Gatan) was operated with a slit width of 20 eV. Automated data acquisition was performed with FEI EPU v2.12.1 software at a nominal magnification of 130,000x, corresponding to a pixel size of 0.654 Å/px. Data acquisition was done with a defocus range of 0.2–1.4 μm. Image stacks of 70 frames were collected over 1 s in counting mode. The total dose for the sample was 70.0 e^−^/Å^2^. A total of 11,484 image stacks were collected. A second data collection from the same grid was done at a 15° tilt to help combat orientation bias. Data acquisition was done with a defocus range of 0.2–1.4 μm. Image stacks of 71 frames were collected over 1 s in counting mode. The total dose for the sample was 70.65 e^−^/Å^2^. A total of 14,087 image stacks were collected.

Each data set was initially separately processed in cryoSPARC v4.7, with later processing in cryoSPARC v5.0.2.^79^ The image stacks were motion corrected and the contrast transfer function was estimated. Particles were picked using two blob pickers: an elliptical blob picker from 50–150 Å and a circular blob picker from 80–110 Å. Particles were extracted at a box size of 352px and Fourier cropped to 176px. Each particle set was individually subjected to 2D classification with the following parameters (all other parameters were left at their default values): batch size per class = 5000, Number of online EM iterations = 50, Force max over poses/shifts = off, Initial uncertainty factor = 1, number of 2D classes = 100, circular mask diameter = 100 Å. Each particle set underwent 2 rounds of 2D classification, removing 2D classes that represented junk classes after every round of classification. Particles were then re-extracted to re-center them and duplicate particles within each data set were removed. Another round of 2D classification was done, and the junk classes were removed. The particles from both data sets were then combined and subjected to two final rounds of 2D classification.

After this final round of 2D classification, the particle stack contained 5,847,708 particles. All particles were subjected to an ab-initio reconstruction with the following parameters (all other parameters were left at their default values): number of ab-initio classes = 10, maximum resolution = 9, initial resolution = 12, initial minibatch size = 300, final minibatch size = 1000, class similarity = 0. A non-uniform refinement job was performed with the particles and volume of the ab-initio reconstruction class that best resembled P-TEFb and that had extra density for the BRD4-PID, which contained 887,702 particles, with the volume as an input low-pass filtered to 12 Å. The refined volume was then subjected to a local refinement with the following parameters (all other parameters were left at their default values: re-center rotations each iteration = true, re-center shifts each iteration = true, force re-do GS split = true, minimize over per-particle scale = true, rotation search ext = 2 deg, shift search ext = 1 Å). The local refinement was done with the output mask from the non-uniform refinement, which encompassed the whole complex. The locally refined volume was then subjected to 3D classification with the following parameters (all other parameters were left at their default values): number of classes = 2, filter resolution = 3 Å, batch size per class = 5000, intial structure low pass filter = 12 Å, force hard classification = true, initialization mode = PCA, number of O-EM epochs = 5. The most occupied class contained 475,526 particles and was subjected to local refinement using the parameters above and the particles were re-extracted at a box size of 352 px. The particles were then used in a local refinement with the following parameters (all other parameters were left at their default values): re-center rotations each iteration = true, re-center shifts each iteration = true, force re-do GS split = true, minimize over per-particle scale = true, rotation search ext = 2 deg, shift search ext = 1 Å. The particle stack was the subjected to another round of 3D classification using the following parameters (all other parameters were left at their default values): number of classes = 2, filter resolution = 3 Å, batch size per class = 5000, initial structure low pass filter = 12 Å, initialization mode = PCA, number of O-EM epochs = 5, Use latent mixing coefficients = true. The most occupied class contained 352,345 particles and was locally refined using the parameters above and then used in 3D classification using the following parameters (all other parameters were left at their default values): number of classes = 2, filter resolution = 3 Å, batch size per class = 5000, initial structure low pass filter = 20 Å, initialization mode = PCA, number of O-EM epochs = 5, Use latent mixing coefficients = true. The most occupied class contained 249,670 particles was subjected to another local refinement followed by reference-based motion correction and another round of local refinement. The particle stack was then imported into Relion-5. 3D auto-refine was performed on the particle stack using the following parameters (all other parameters were left at their default values): Initial low pass filter = 15 Å, Mask diameter = 200 Å, Blush regularization = ON, Initial angular sampling = 3.7 degrees, Local searches from auto-sampling = 1.8 degrees, resulting in Map 1. Map 1 was then sharpened using a b-factor of −254 and used for model building, resulting in Map 2.

### Model building

Each chain of an AlphaFold3 model of the P-TEFb-PID complex was rigid body fit into Map 2 using ChimeraX. To improve the model fit and to remove low confidence parts of the model, the model was subjected to Phenix v1.20.1–4487 dock_and_rebuild. The model was then flexibly fit into map 2 using the online Namdinator server using default parameters at an expected resolution of 5 Å.^80^ ATP*γ*S was modelled by aligning a previously determined structure of human CDK7 bound to ATP*γ*S (PDB 8P6Y) onto our model, extracting the coordinates for ATP*γ*S from this model, and integrating the ATP*γ*S coordinates into our model.^81^ The model was manually adjusted in Coot v1.3.1^82^ and was real-space refined in Phenix v1.20.1–4487.^83^ ReadySet was used to prepare input PDB models for refinement. Real space refinement was performed with global minimization, occupancy, nqh flips, local grid search, and adp on, with max iterations of 1000 and 10 macro cycles, and a nonbonded weight of 1000. Data collection and modeling statistics are in Table 1.

### Multiple Sequence Alignment

Sequences for CDK9, BRD4, and AFF4 were obtained from UniProt. Sequences were aligned using MAFFT v7 online server and visualized in JalView v2.11.5.1.

## Supplementary Material

1

## Figures and Tables

**Figure 1.**
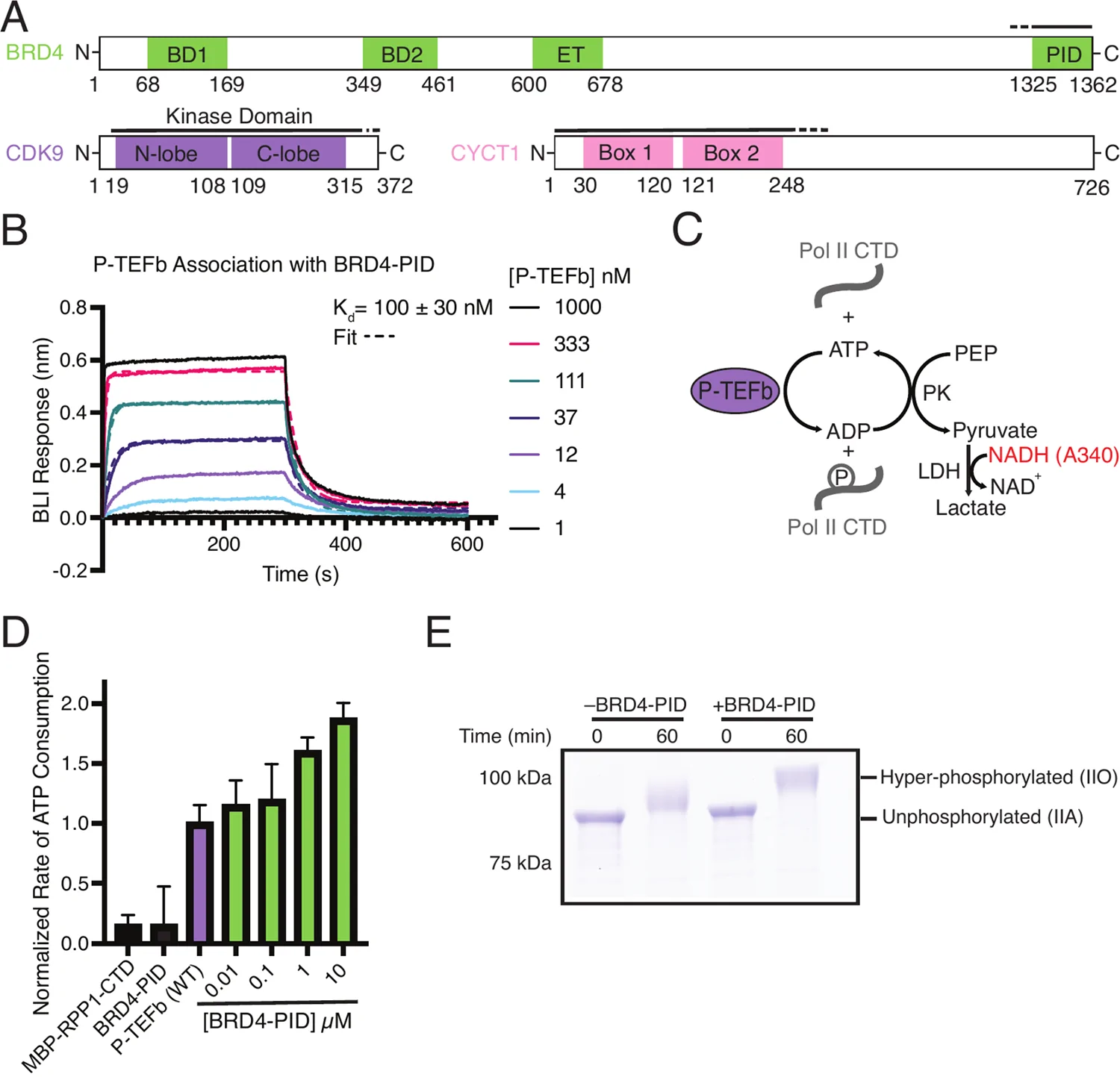
BRD4-PID binds and stimulates P-TEFb activity. (A) Domain architecture of BRD4 (green), CDK9 (purple), and CYCT1 (pink) with residue numbers indicating domain boundaries. Solid lines indicate the regions of the protein modelled in cryo-EM reconstructions reported here. Dashed lines indicate the regions of protein that were not modeled in our structure but are in our purified protein constructs. (B) P-TEFb binding to BRD4-PID as detected by Biolayer interferometry (BLI). Concentration ranges shown. K_d_ was calculated from concentrations 333 nM, 111 nM, and 37 nM. Dashed lines for those concentrations indicate the fit used to calculate the K_d_. K_d_ was calculated from an average of three replicates (replicates shown in Figure S1C) and is 100 nM ± 30 nM. (C) Schematic showing the modified NADH-coupled ATPase assay used to test P-TEFb kinase activity. (D) P-TEFb kinase activity measured using the NADH-coupled ATPase assay. Bars correspond to the normalized change in absorbance at 340 nm as a function of time relative to P-TEFb kinase activity in the presence of MBP-RPB1-CTD. Each bar corresponds to the mean of three individual experiments. BRD4-PID titration shown, as well as the background signal from MBP-RPB1-CTD and BRD4-PID. Error bars represent the standard deviation between three individual experiments. (E) Gel based assay to detect phosphorylation of MBP-RPB1-CTD by SDS-PAGE. P-TEFb was incubated with and without BRD4-PID for one hour. Addition of phospho-groups onto MBP-RPB1-CTD cause the protein to migrate slower in SDS-PAGE due to the increase in negative charges. The positions of unphosphorylated (IIA) and hyper-phosphorylated (IIO) MBP-RPB1-CTD are indicated.

**Figure 2.**
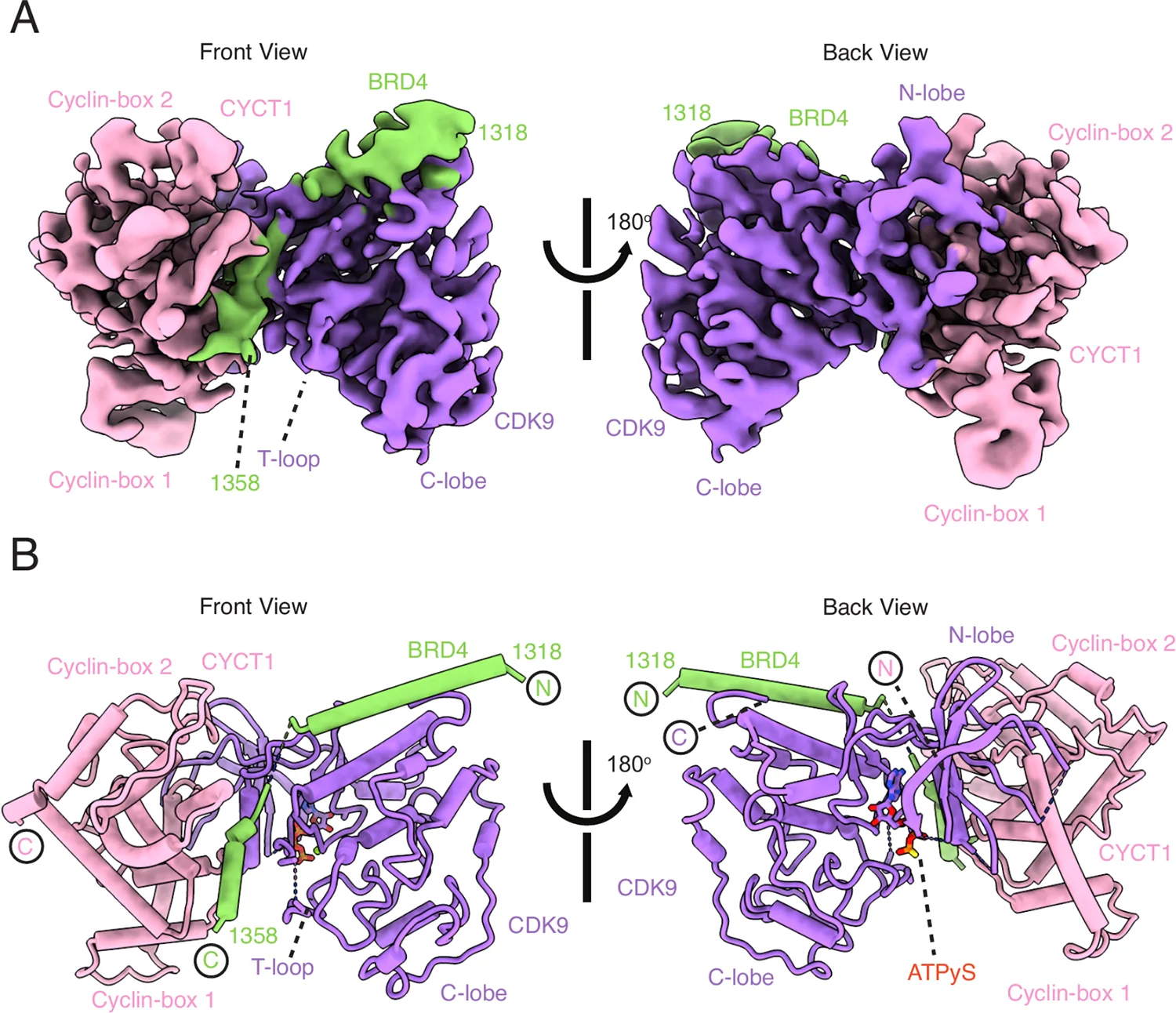
Structure of the P-TEFb–BRD4-PID complex. (A) Cryo-EM density map of the P-TEFb–BRD4-PID complex. BRD4 green, CYCT1 pink, CDK9 purple. Front and back views are shown. (B) Structure of the P-TEFb–BRD4-PID complex. Same coloring and views as in (A).

**Figure 3.**
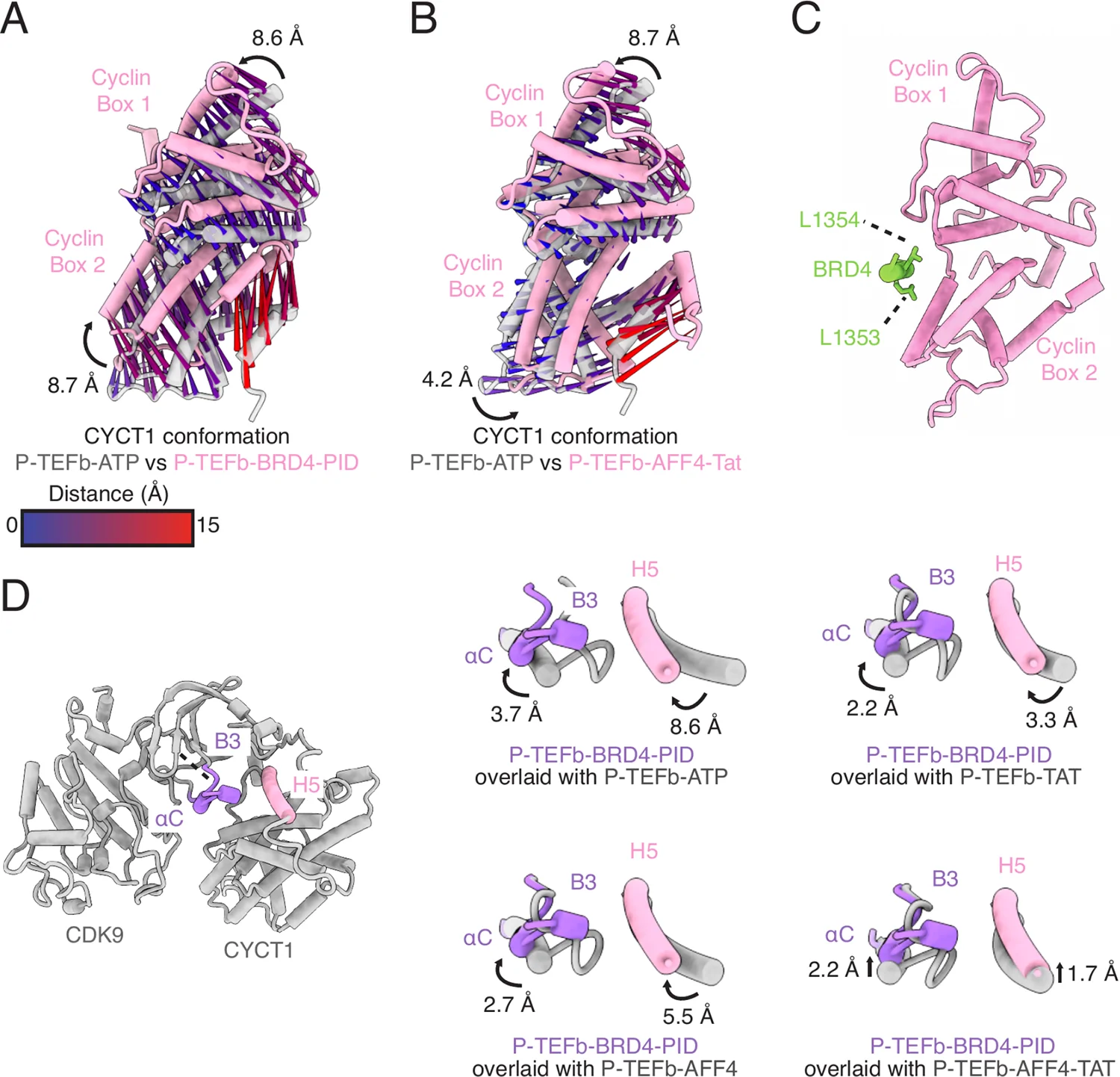
Structural rearrangements upon transcription factor binding to P-TEFb. (A) Overlay of CYCT1 when P-TEFb is bound to BRD4 (pink) or ATP (grey) (PDB ID: 3BLQ). Structures aligned on CDK9. Structures rotated 60° along the x-axis from the back view. Trajectories show the relative difference in amino acid positioning from the ATP bound state to the BRD4-PID bound state. Trajectories colored based on distance: blue (0 Å) to red (15 Å). Distances calculated for CYCT1 residues 124 and 163. (B) Overlay of CYCT1 when P-TEFb is bound to AFF4–Tat (pink) or ATP (grey) (PDB ID: 3BLQ). Views and coloring the same as in (A). (C) Leucine 1353 and 1354 interact with CYCT1. Residues shown as spheres. Views and coloring the same as in (A). (D) CDK9 αC and CYCT1 H5 rearrangements. Left: Position of the CDK9 αC-B3 linker (purple) and CYCT1 H5 (pink) relative to P-TEFb–BRD4-PID model (gray). BRD4-PID not shown for clarity. Right: conformational changes for the αC-B3 linker (purple) and CYCT1 H5 (pink) elements in the BRD4-PID bound state relative to P-TEFb bound to ATP (PDB ID: 3BLQ), P-TEFb–Tat (PDB ID: 3MIA), P-TEFb–AFF4 (4IMY), and P-TEFb–AFF4–Tat (4OGR), all colored in grey. Distances measured from CDK9 amino acid 61 and CYCT1 amino acid 124, respectively.

**Figure 4.**
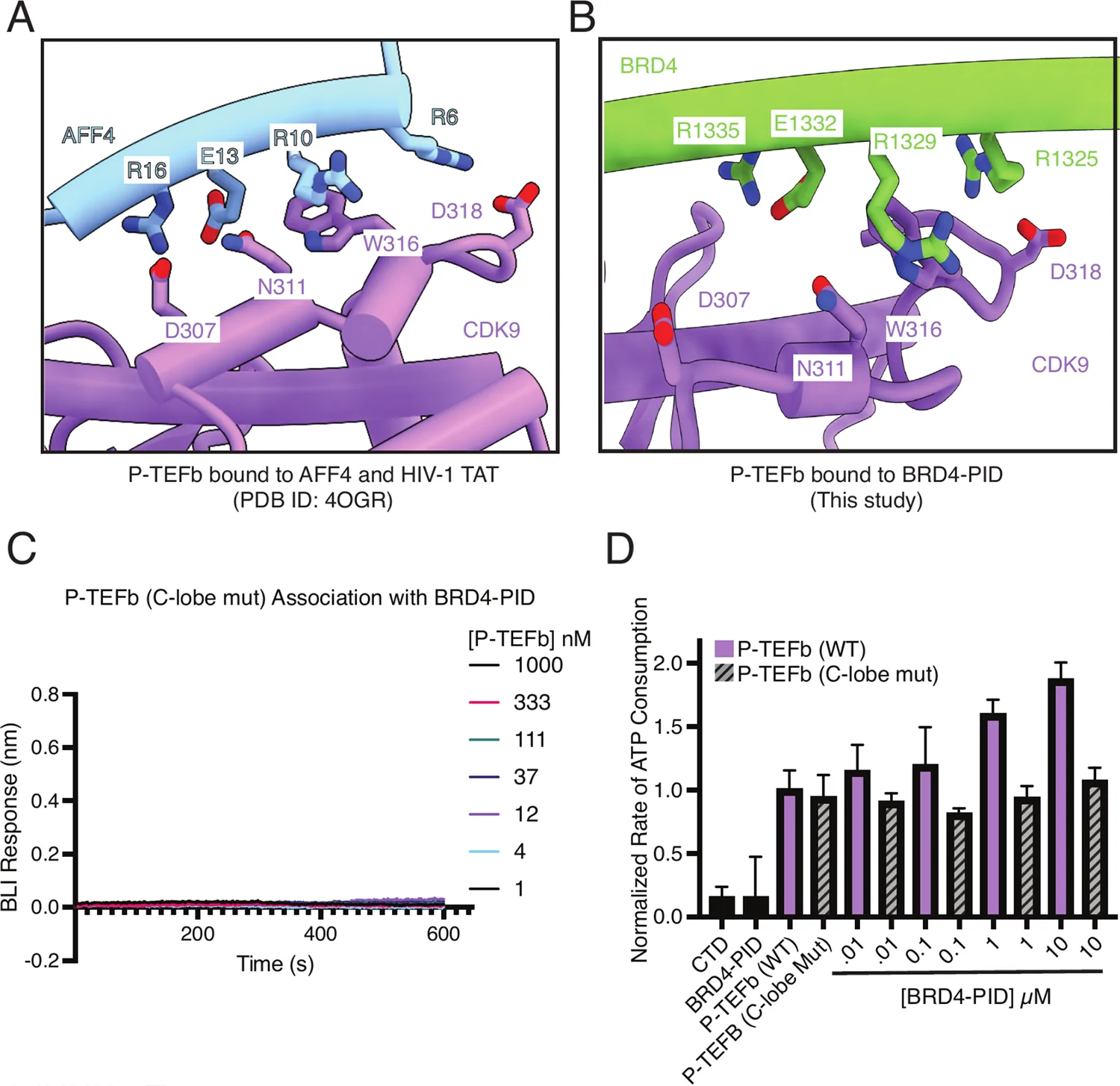
BRD4 and AFF4 interact with the C-lobe of P-TEFb. (A) Interaction between BRD4 (green) and CDK9’s C-lobe (purple). Key residues for the interaction shown as spheres. (B) Interaction between AFF4 (blue) and CDK9’s C-lobe (purple) (PDB ID: 4OGR). Key residues for the interaction are shown. (C) P-TEFb (C-lobe mutant) binding to BRD4-PID as detected by Biolayer interferometry (BLI). Concentration ranges shown. (D) P-TEFb kinase activity measured using the NADH-coupled ATPase assay. Bars correspond to the normalized change in absorbance at 340 nm as a function of time relative to P-TEFb kinase activity in the presence of MBP-RPB1-CTD and BRD4 titration to P-TEFb shown, as well as the background signal from MBP-RPB1-CTD and BRD4-PID are shown. Error bars represent the standard deviation between three individual experiments. Each bar corresponds to the mean of three individual experiments.

**Figure 5.**
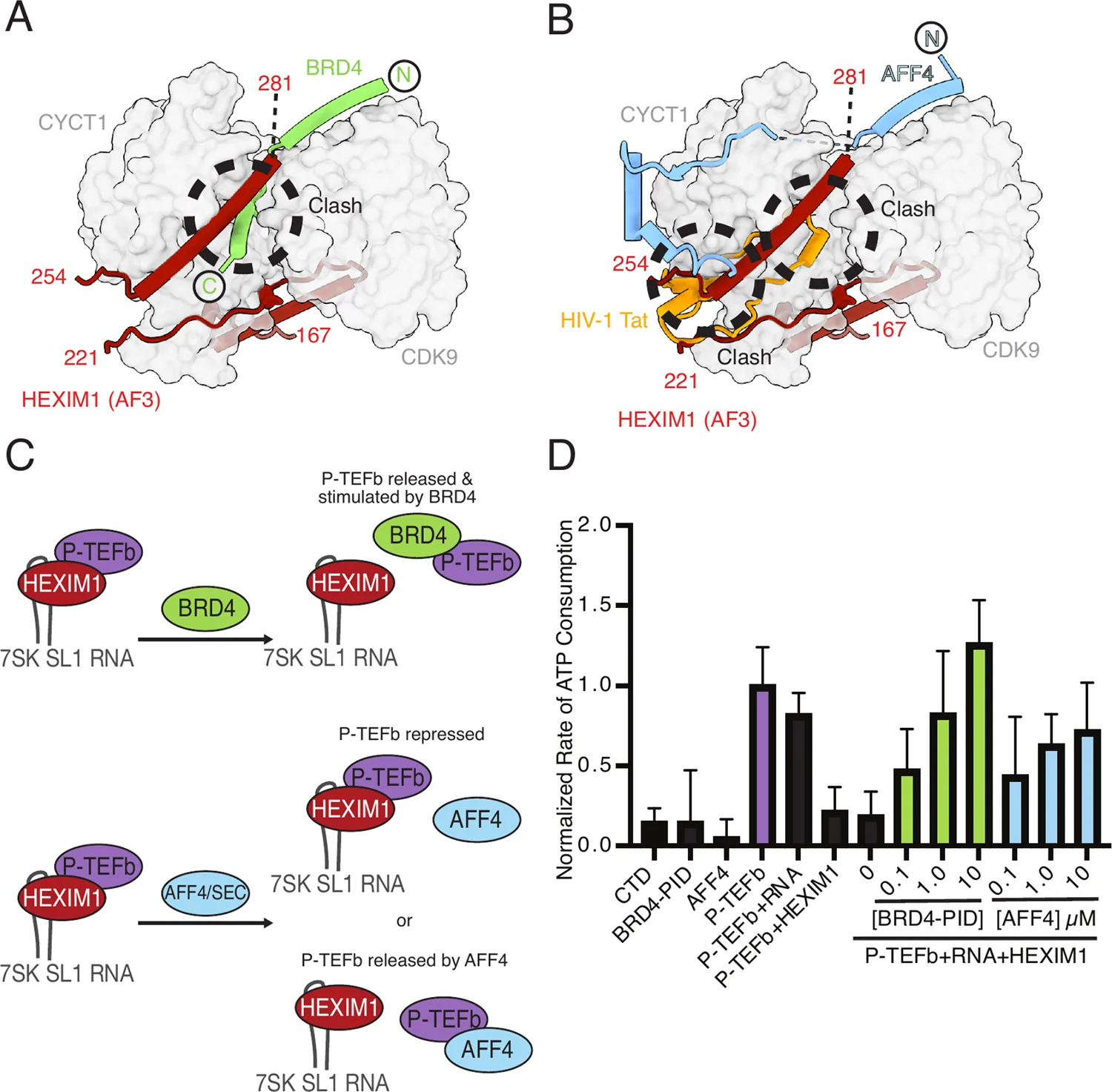
BRD4 and AFF4 release P-TEFb from the inhibited 7SK RNP complex. (A) AlphaFold 3 model of HEXIM1 bound to P-TEFb overlaid with our model of P-TEFb bound to BRD4-PID. P-TEFb shown as a gray surface. HEXIM1 shown in red and BRD4-PID shown in green. Location of HEXIM1 and BRD4-PID clash is shown. (B) AlphaFold 3 model of HEXIM1 bound to P-TEFb overlaid with the structure of P-TEFb bound to AFF4–Tat shown. P-TEFb shown as a gray surface. HEXIM1 shown in red and AFF4 shown in blue and Tat orange. Location where HEXIM1 clashes with AFF4 and Tat are shown. (C) Cartoon illustrating the role that BRD4 and AFF4 play on P-TEFb activity in the context of the 7SK RNP complex. (D) P-TEFb kinase activity measured using the NADH-coupled ATPase assay. Bars correspond to the normalized change in absorbance at 340 nm as a function of time relative to P-TEFb kinase activity in the presence of MBP-RPB1-CTD. P-TEFb incubated with RNA and RPB1-CTD and P-TEFb incubated with HEXIM1 and RPB1-CTD shown. RPB1-CTD, as well as BRD4-PID and AFF4 (1–98) incubated with RPB1-CTD are also shown. P-TEFb incubated with RNA and HEXIM1 is shown. BRD4-PID and AFF4 (1–98) were titrated into the P-TEFb–HEXIM1–RNA complex. Error bars represent the standard deviation between three individual experiments. Each bar corresponds to the mean of three individual experiments.

**Figure 6.**
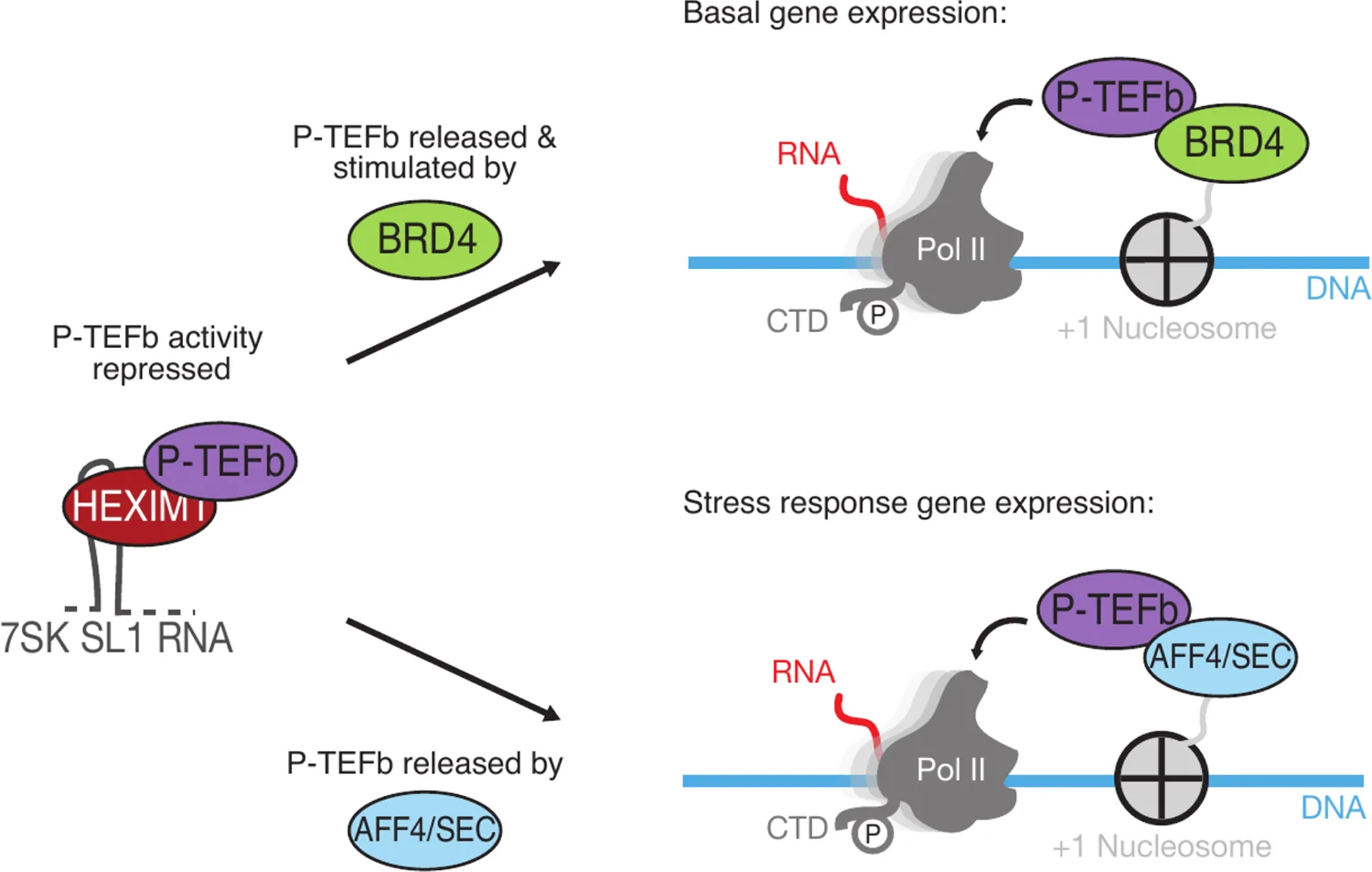
Model of P-TEFb’s role in transcription. (A) Cartoon showing that P-TEFb activity is repressed upon binding to HEXIM1 as a part of the 7SK RNP complex. BRD4 or AFF4 can release P-TEFb from the 7SK RNP complex, and recruit P-TEFb to RNA Pol II, leading to productive elongation.

**Table 1.** Cryo-EM data collection, refinement, and validation statistics.

	P-TEFb-BRD4-PID Complex
	(EMD-XXXXX)
	(PDB: XXXX)
Data collection and processing	
Magnification	130,000
Voltage (kV)	300
Electron exposure (e-/Å2)	70.0, 70.65
Defocus range (μm)	0.2–1.4
Pixel size (Å)	0.654
Symmetry imposed	C1
Initial particle images (no.)	5,847,708
Final particle images (no.)	238,413
Map resolution (Å)	4.3
FSC threshold	0.143
Map resolution range (Å)	4.0–5.0
Refinement	
Initial models used (PDB code)	AlphaFold 3 Model
Map sharpening B factor (Å2)	−245
Model composition	
Non-hydrogen atoms	4616
Protein residues	562
Nucleotides	0
Ligands	AGS: 1
B factors (Å2)	
Protein	73.15
Nucleotide	0
Ligand	75.37
R.m.s. deviations	
Bond lengths (Å)	0.004
Bond angles (°)	0.888
Validation	
MolProbity score	1.71
Clashscore	7.02
Poor rotamers (%)	0.00
Ramachandran plot	
Favored (%)	95.42
Allowed (%)	4.58
Disallowed (%)	0.00

## Data Availability

Cryo-EM maps have been deposited in the Electron Microscopy Data Bank under accession code EMD-[XXXXX]. Atomic coordinates have been deposited in the Protein Data Bank under accession code [XXXX]. All other data supporting the findings of this study are available within the article and its supplementary information.

## SIGNIFICANCE STATEMENT

The exquisite control of gene expression is required for proper development and cellular response to environmental stress. Positive transcription elongation factor b (P-TEFb) is required for the phosphorylation of RNA Pol II and leads to the coordinated release of RNA Pol II from pause sites near the promoter. P-TEFb is bound to bromo-domain containing protein 4 (BRD4), which stimulates P-TEFb activity; however, it is unknown how BRD4 associates with P-TEFb. Here we report a cryo-EM structure of P-TEFb bound to BRD4, revealing how BRD4 interacts with P-TEFb. Comparison of our structure with previously reported P-TEFb structures shows how P-TEFb is regulated by different transcription factor binding partners.
