# Supplementary material for: Structural basis for P-TEFb association with BRD4
Source: bioRxiv. 2026 Sep 7:2026.09.03.749171. Preprint. [Version 1] doi: 10.64898/2026.09.03.749171 (PMC13622488; doi:10.64898/2026.09.03.749171)
Supplement: Supplementary file 1 [file NIHPP2026.09.03.749171V1-supplement-1.pdf]

bioRxiv preprint doi: https://doi.org/10.64898/2026.09.03.749171; this version posted September 7, 2026. The copyright holder for this preprint (which was not certified by peer review) is the author/funder, who has granted bioRxiv a license to display the preprint in perpetuity. It is made available under a CC-BY 4.0 International license.

SUPPLEMENTARY FIGURES

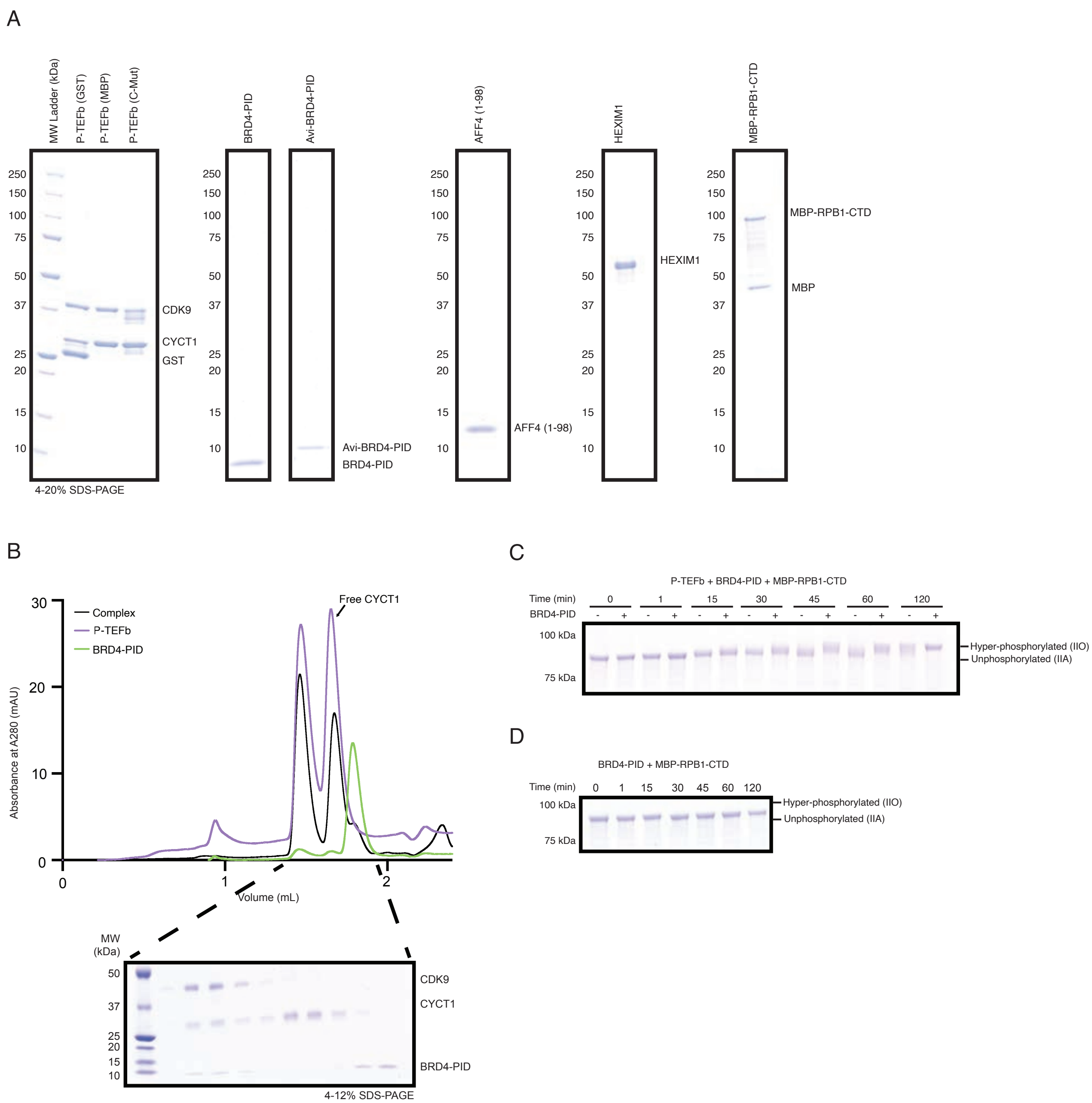

Supplementary Figure 1. Proteins used in this study and biochemical assays characterizing P-TEFb-BRD4-PID interaction.

(A) Coomassie stained gels of purified proteins used in this study. 5 µM of protein loaded per well. Molecular weight markers are shown.

(B) Analytical size exclusion chromatography traces of the P-TEFb–BRD4 complex (black), P-TEFB alone (purple), and BRD4-PID alone (green). Coomassie stained gel of the peak fractions of the complex shown. Molecular weight markers are shown.

(C) Full-time course titration of P-TEFb stimulation of RPB1-CTD phosphorylation by BRD4-PID. Coomassie stained gel showing the differential migration of MBP-RPB1-CTD as P-TEFb phosphorylates the protein. Molecular weight markers are shown. The experiment was performed 3 times.

(D) Full-time course titration of BRD4-PID incubated with RPB1-CTD. Coomassie stained gel showing that the migration of MBP-RPB1-CTD does not change with the incubation of just BRD4-PID. Molecular weight markers are shown. The experiment was performed 3 times.

bioRxiv preprint doi: https://doi.org/10.64898/2026.09.03.749171; this version posted September 7, 2026. The copyright holder for this preprint (which was not certified by peer review) is the author/funder, who has granted bioRxiv a license to display the preprint in perpetuity. It is made available under a CC-BY 4.0 International license.

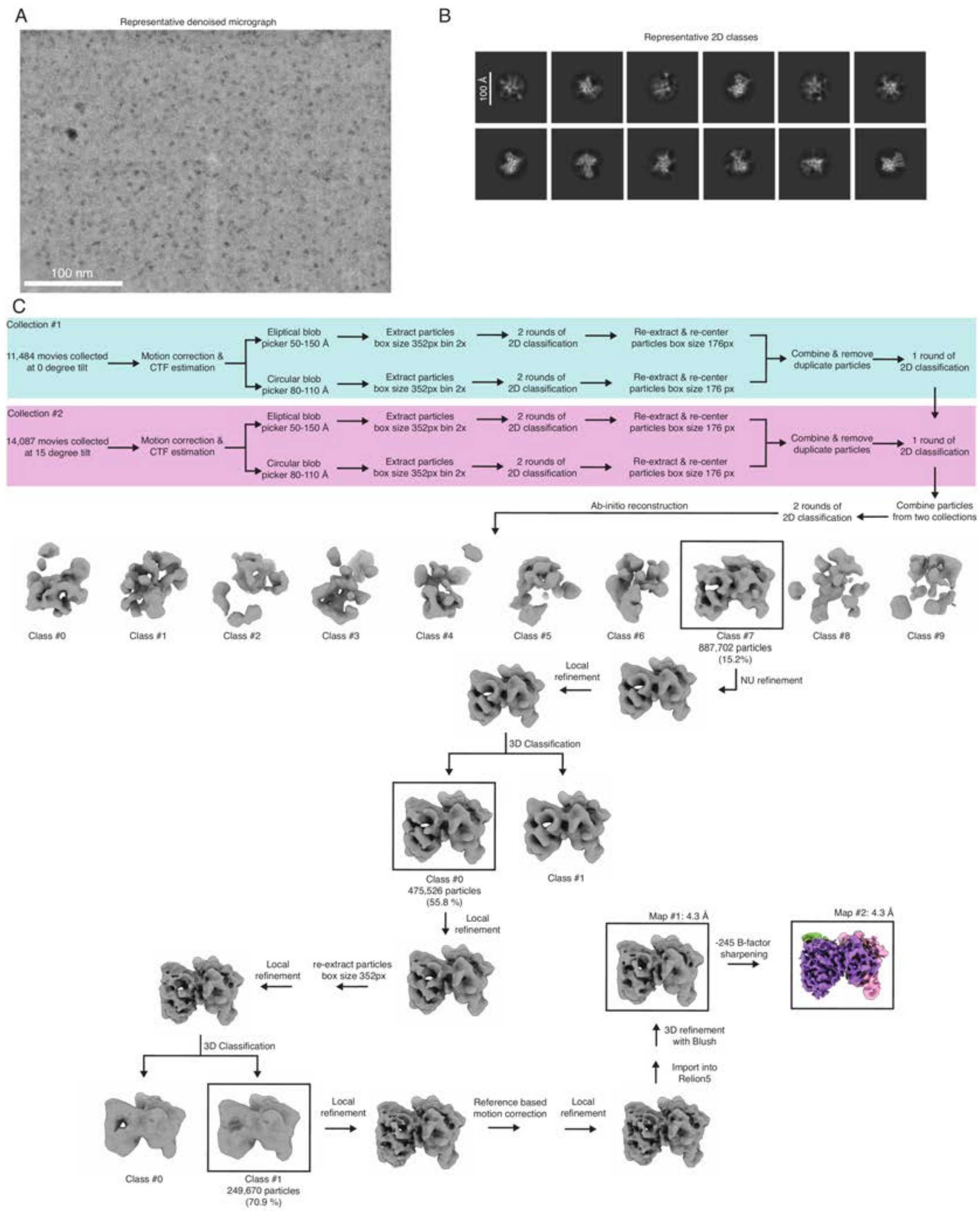

Supplementary Figure 2. Cryo-EM data processing.
(A) Representative denoised micrograph.
(B) Representative 2D classes.
(C) Cryo-EM data processing tree for the P-TEFb-BRD4-PID complex.

bioRxiv preprint doi: https://doi.org/10.64898/2026.09.03.749171; this version posted September 7, 2026. The copyright holder for this preprint (which was not certified by peer review) is the author/funder, who has granted bioRxiv a license to display the preprint in perpetuity. It is made available under a CC-BY 4.0 International license.

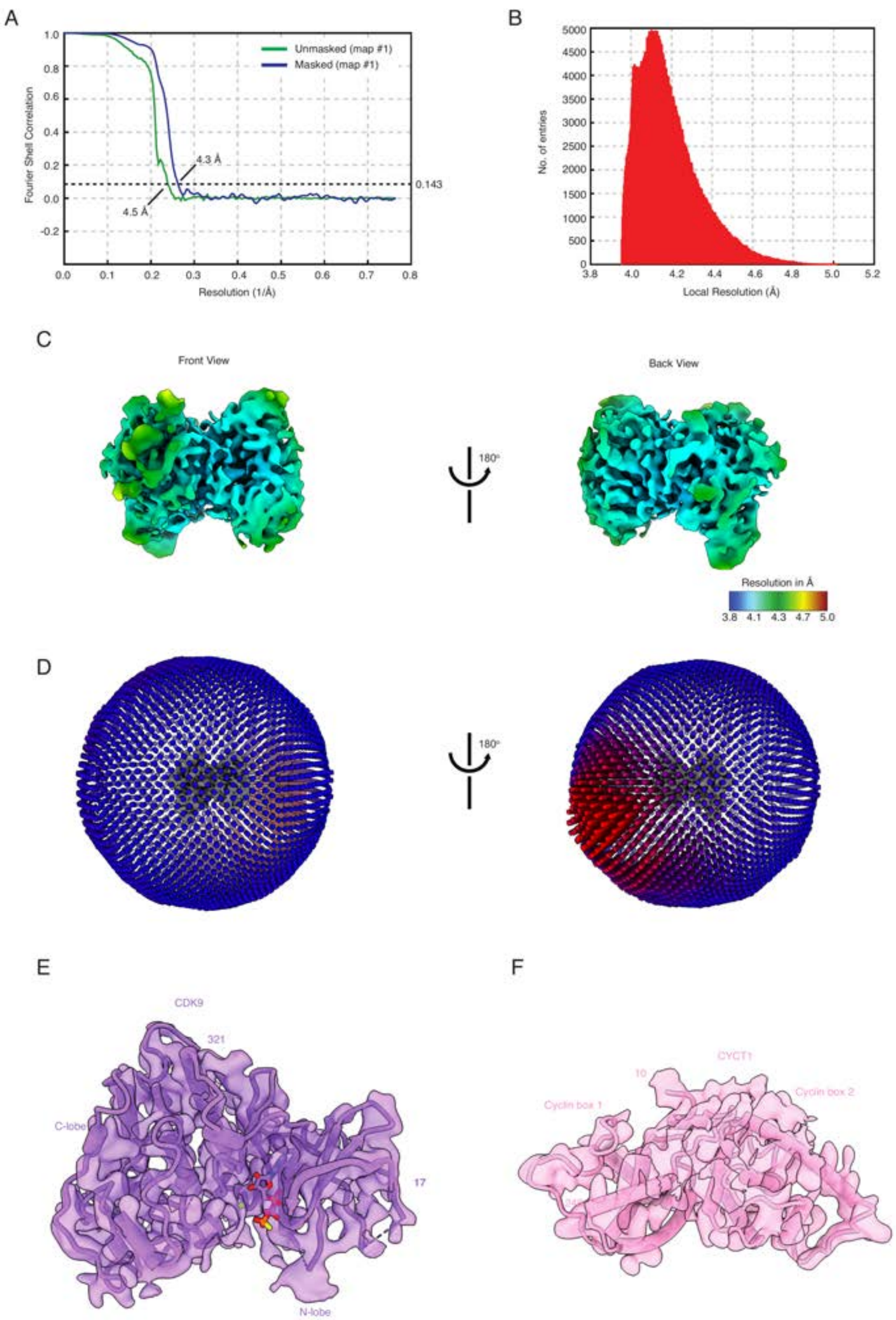

Supplementary Figure 3. Cryo-EM data statistics.
(A) Fourier Shell Correlation (FSC) plots for masked and unmasked maps.
(B) Histogram of local resolution.
(C) Front and back views of map #2 colored by local resolution.
(D) Front and back views of map #2 with orientation distributions.
(E) Representative density and model for CDK9 (map #2).
(F) Representative density and model for CYCT1 (map #2).

bioRxiv preprint doi: https://doi.org/10.64898/2026.09.03.749171; this version posted September 7, 2026. The copyright holder for this preprint (which was not certified by peer review) is the author/funder, who has granted bioRxiv a license to display the preprint in perpetuity. It is made available under a CC-BY 4.0 International license.

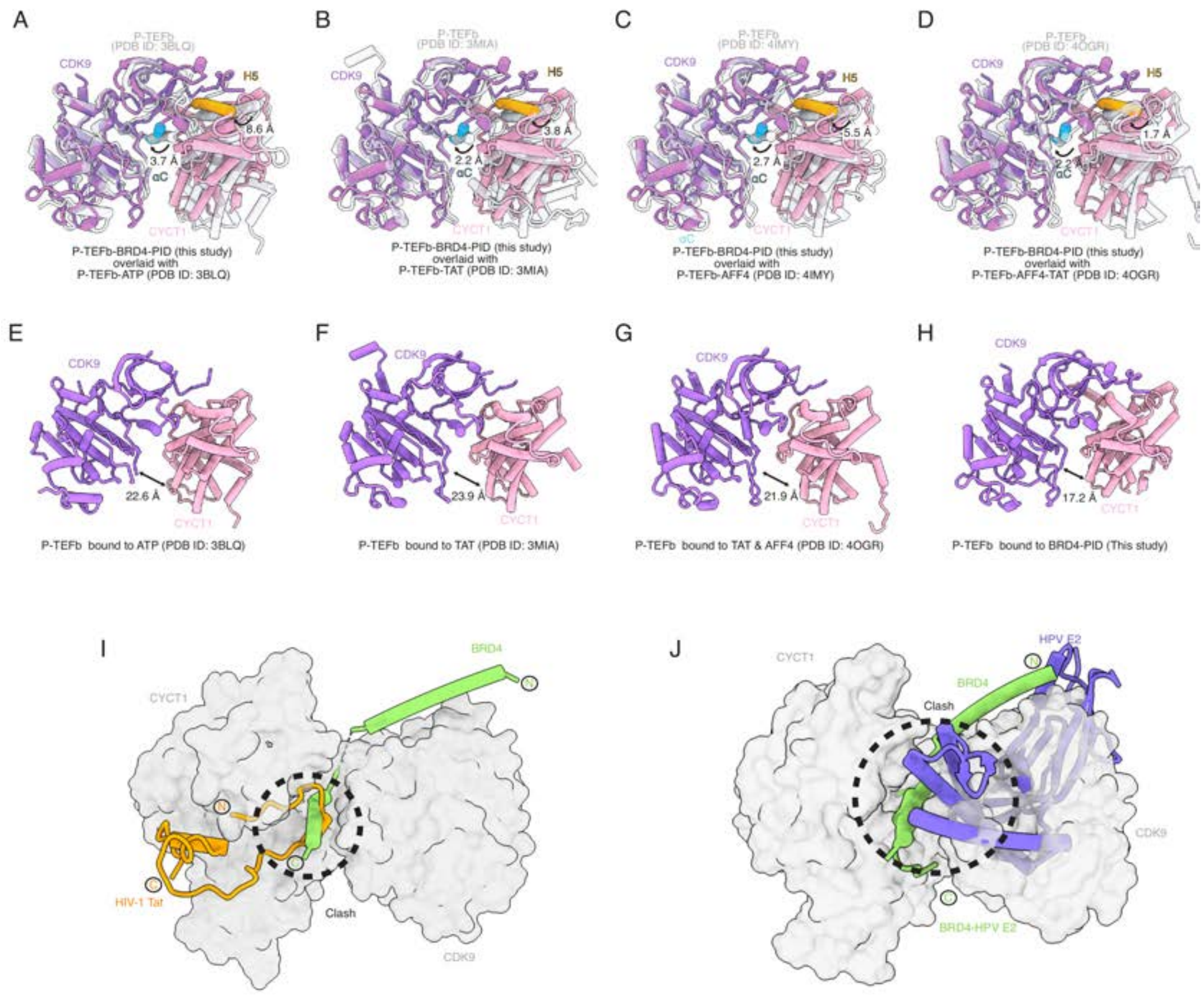

Supplementary Figure 4. Comparison of P-TEFb structures and mutant binding data.
(A-D) Complete structural alignment of P-TEFb-BRD4 to (A) P-TEFb bound to ATP (PDB ID: 3BLQ), (B) with P-TEFb bound to Tat (PDB ID: 3MIA), (C) P-TEFb bound to AFF4 (PDB ID: 4IMY), and (D) P-TEFb bound to AFF4–HIV1-Tat (PDB ID: 4OGR). CDK9 and CYCT1 are only shown for clarity. CDK9 (purple) and CYCT1 (pink) from the P-TEFb-BRD4 model. All other P-TEFb models shown in grey. CDK9 αC shown in blue and CYCT1 H5 shown in yellow. Measurements as in **Figure 3**.
(E) Model of P-TEFb bound to ATP. CDK9 is shown in purple and CYCT1 is shown in pink. Measurement between CDK9 (residue 175) and CYCT1 (residue 163) shown.
(F) Model of P-TEFb bound to Tat. CDK9 is shown in purple and CYCT1 is shown in pink. Measurement between CDK9 (residue 175) and CYCT1 (residue 163) shown.
(G) Model of P-TEFb bound to AFF4 and Tat. CDK9 is shown in purple and CYCT1 is shown in pink. Measurement between CDK9 (residue 175) and CYCT1 (residue 163) shown.
(H) Model of P-TEFb bound to BRD4-PID. CDK9 is shown in purple and CYCT1 is shown in pink. Measurement between CDK9 (residue 175) and CYCT1 (residue 163) shown.
(I) HIV-1 Tat (orange) (PDB ID: 3MIA) modeled onto P-TEFb (gray in surface) bound to BRD4-PID (green) model. Clash between Tat and BRD4-PID indicated.
(J) HPV E2 (blue) (PDB ID: 2NNU) modeled onto the P-TEFb (gray in surface) bound to BRD4-PID (green) model. Clash between E2 and BRD4-PID is indicated.

bioRxiv preprint doi: https://doi.org/10.64898/2026.09.03.749171; this version posted September 7, 2026. The copyright holder for this preprint (which was not certified by peer review) is the author/funder, who has granted bioRxiv a license to display the preprint in perpetuity. It is made available under a CC-BY 4.0 International license.

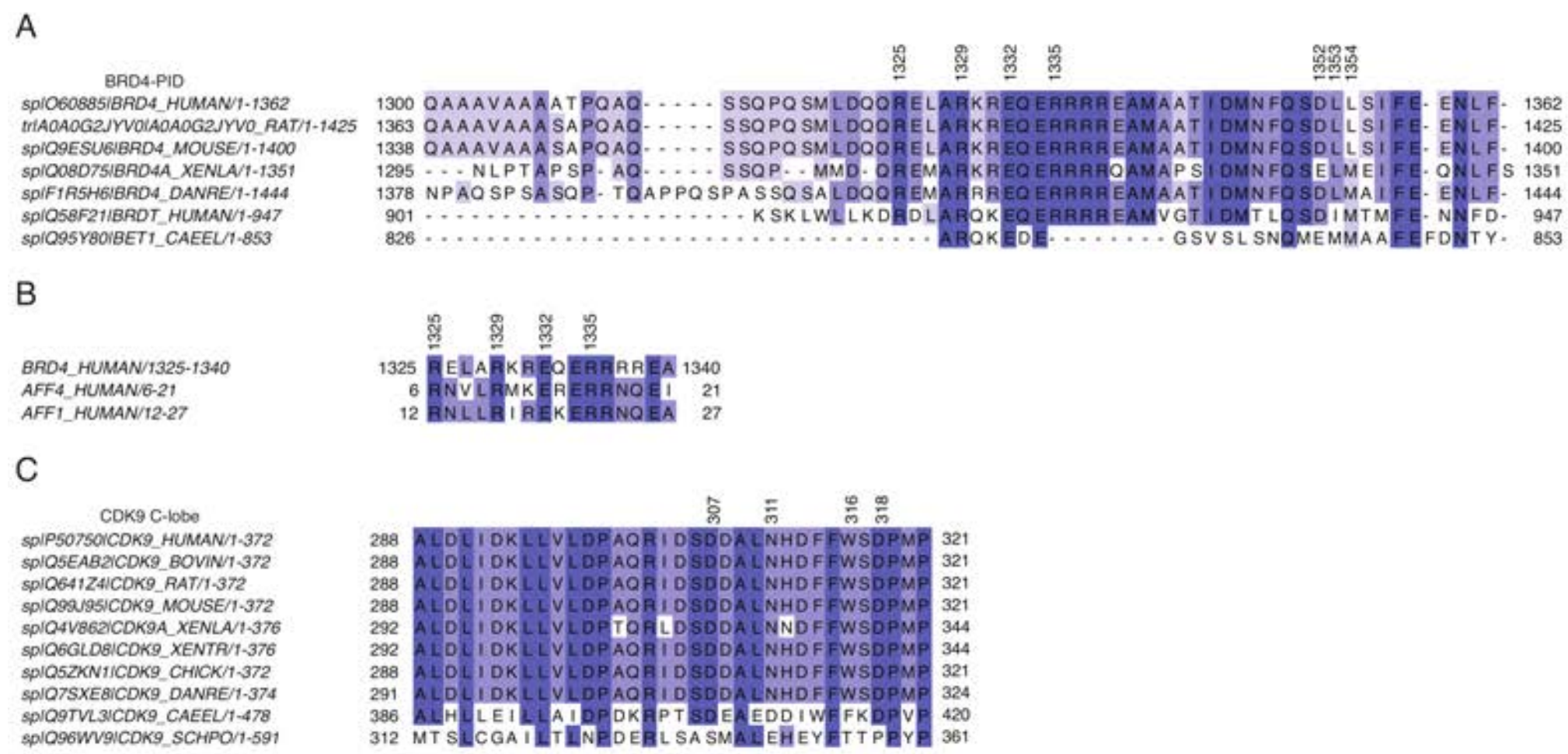

Supplementary Figure 5. Sequence Alignments for CDK9, CDK7, and BRD4.
(A) Sequence alignment for BRD4 residues 1300–1362.
(B) Sequence alignment for BRD4 residues 1325–1340, AFF4 residues 6–21, and AFF4 residues 12–27.
(C) Sequence alignment for CDK9 residues 288–321.

bioRxiv preprint doi: https://doi.org/10.64898/2026.09.03.749171; this version posted September 7, 2026. The copyright holder for this preprint (which was not certified by peer review) is the author/funder, who has granted bioRxiv a license to display the preprint in perpetuity. It is made available under a CC-BY 4.0 International license.

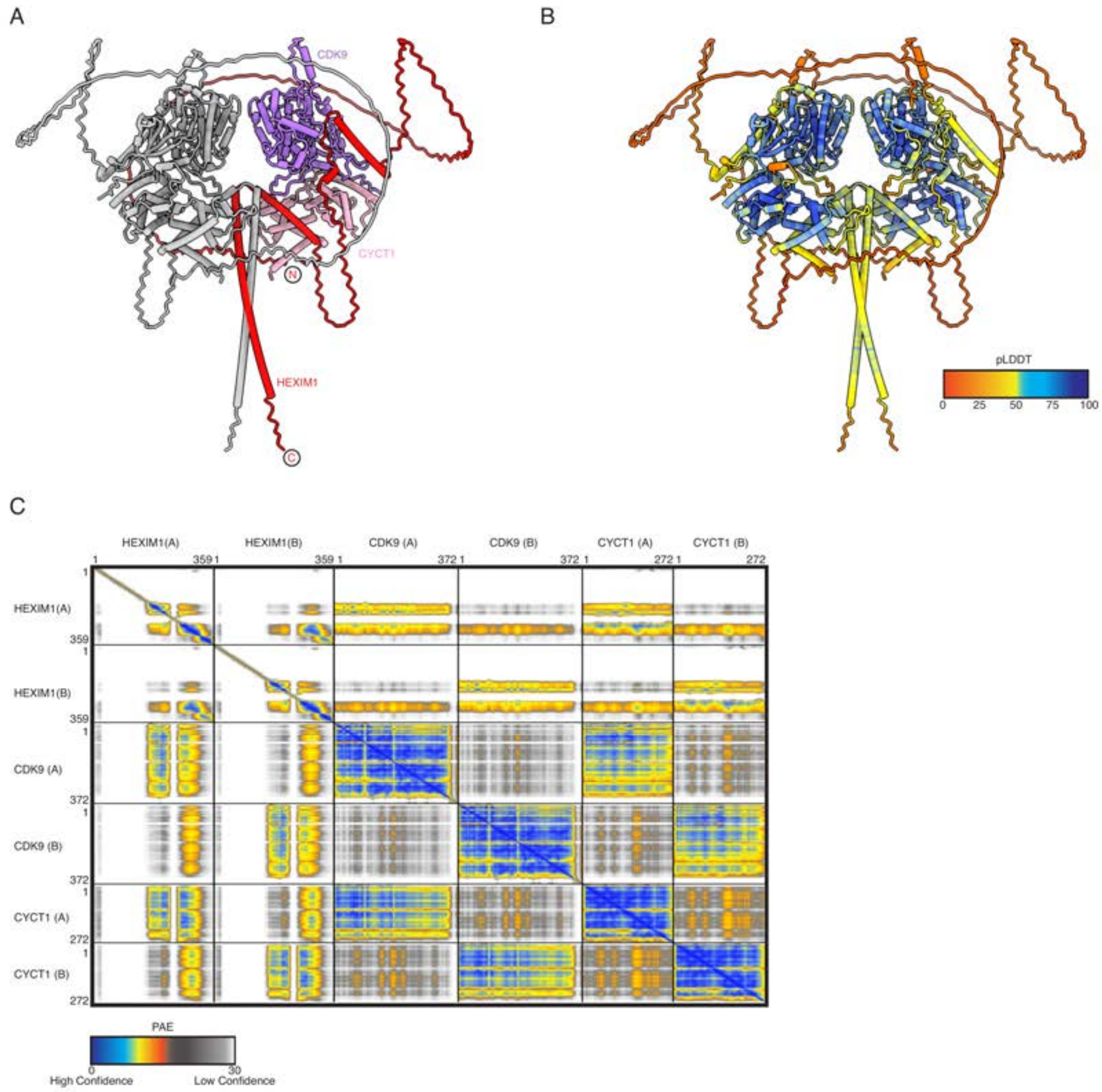

Supplementary Figure 6. AlphaFold 3 modeling for HEXIM1–P-TEFb complex.
(A) P-TEFb–HEXIM1 dimer shown with one half of the dimer colored in gray and the other half colored with CDK9 in purple, CYCT1 in pink, and HEXIM1 in red.
(B) P-TEFb–HEXIM1 dimer colored with the pLDDT scores, scale bar shown.
(C) P-TEFb–HEXIM1 prediction PAE plot, scale bar shown.
